# Supplementary material for: The Graph of Our Mind
Source: Brain Sci. 2021 Mar 8;11(3):342. doi: 10.3390/brainsci11030342 (PMC7998275; doi:10.3390/brainsci11030342)
Supplement: Supplementary file 1 [file brainsci-11-00342-s001.pdf]

Supporting Material for the Article: “The Graph of Our Mind”

Balázs Szalkai[a], Bálint Varga[a], Vince Grolmusz[a,b,*]

[a] *PIT Bioinformatics Group, Eötvös University, H-1117 Budapest, Hungary*
[b] *Uratim Ltd., H-1118 Budapest, Hungary*

A common remark for all tables: p-values, less than 0.00001 are rounded up to 0.00001 uniformly, since we did not want to use floating point numbers in the tables.

Table S1:

The results and the statistical analysis of the graph-theoretical evaluation of the sex differences in the 426-subject data set. The first column gives the resolutions: the number of vertices in the whole graph. The second column describes the graph parameter computed: its syntactics is as follows: each parameter-name contains two separating “_” symbols that define three parts of the parameter-name. The first part describe the hemisphere or the whole connectome with the words Left, Right or All. The second part describes the parameter computed, and the third part the weight function used. The third column contains values of the parameters, averaged to the sexes. The fourth column describes the p-values of the first round, the fifth column the p-values of the second round, and the sixth column the (very strict) Holm-Bonferroni correction of the p-value. With p=0.05 *all* the rows with boldface last column describe significantly different graph theoretical properties between sexes. One-by-one, each row with italic fifth column describe significant differences between sexes, with p=0.05. For the details, we refer to the section “Statistical analysis”.

**Scale**	**Property**	**Female | Male**	**p (1st)**	**p (2nd)**	**p (corrected)**
129	Left_PGEigengap_FiberNDivLength	0.0948|0.0811	0.00001	*0.00001*	**0.00001**
234	Left_PGEigengap_FiberNDivLength	0.0712|0.0606	0.00001	*0.00001*	**0.00001**
129	Left_PGEigengap_FiberN	0.1219|0.1007	0.00001	*0.00001*	**0.00001**
83	Left_PGEigengap_FiberNDivLength	0.1412|0.1249	0.00001	*0.00001*	**0.00001**
234	Left_PGEigengap_FiberN	0.0946|0.0782	0.00001	*0.00001*	**0.00001**
83	Left_PGEigengap_FiberN	0.1675|0.1430	0.00001	*0.00001*	**0.00001**
234	All_PGEigengap_FiberNDivLength	0.0242|0.0201	0.00001	*0.00001*	**0.00001**
83	Left_MinCutBalDivSum_FiberNDivLength	0.1320|0.1186	0.00001	*0.00001*	**0.00001**
83	All_LogSpanningForestN_FiberNDivLength	147.7706|142.7239	0.00001	*0.00001*	**0.00001**
83	Left_MinCutBalDivSum_FiberN	0.1305|0.1151	0.00001	*0.00001*	**0.00001**
129	All_PGEigengap_FiberNDivLength	0.0284|0.0237	0.00001	*0.00001*	**0.00001**
83	All_Sum_FiberN	11072.8196|10547.3855	0.00001	*0.00001*	**0.00001**
129	Left_MinCutBalDivSum_FiberN	0.1223|0.1052	0.00001	*0.00001*	**0.00001**
83	All_PGEigengap_FiberNDivLength	0.0346|0.0291	0.00001	*0.00001*	**0.00001**
83	Left_Sum_Unweighted	282.0573|269.7710	0.00001	*0.00001*	**0.00001**
234	Left_MinCutBalDivSum_FiberN	0.0995|0.0864	0.00001	*0.00001*	**0.00002**
83	All_Sum_FAMean	218.7173|202.2306	0.00001	*0.00001*	**0.00002**
463	Left_MinCutBalDivSum_FiberN	0.0702|0.0608	0.00001	*0.00001*	**0.00002**
129	All_Sum_FiberN	12238.966|11779.5060	0.00001	*0.00001*	**0.00003**
83	Left_LogSpanningForestN_FiberNDivLength	73.9377|71.1251	0.00001	*0.00001*	**0.00003**

*Corresponding author

Email addresses: szalkai@pitgroup.org (Balázs Szalkai), varga@pitgroup.org (Bálint Varga), grolmusz@pitgroup.org (Vince Grolmusz)

234	Left_PGEigengap_Unweighted	0.1282|0.1104	0.00001	*0.00001*	**0.00004**
83	All_LogSpanningForestN_FAMean	109.3931|102.6911	0.00001	*0.00001*	**0.00005**
83	All_Sum_Unweighted	564.4098|544.3012	0.00001	*0.00001*	**0.00006**
83	Left_Sum_FAMean	105.9875|97.2824	0.00001	*0.00001*	**0.00006**
129	Left_PGEigengap_Unweighted	0.2047|0.1774	0.00001	*0.00001*	**0.00006**
463	Left_MinCutBalDivSum_Unweighted	0.0927|0.0805	0.00001	*0.00001*	**0.00007**
234	All_PGEigengap_FiberN	0.0250|0.0212	0.00001	*0.00001*	**0.00007**
129	All_LogSpanningForestN_FiberNDivLength	210.3350|204.5640	0.00001	*0.00001*	**0.00007**
83	Left_LogSpanningForestN_FAMean	53.1346|49.1865	0.00001	*0.00001*	**0.00008**
83	Left_PGEigengap_Unweighted	0.3083|0.2769	0.00001	*0.00001*	**0.00010**
83	Left_MinCutBalDivSum_FAMean	0.24907|0.2279	0.00001	*0.00001*	**0.00013**
129	Left_PGEigengap_FAMean	0.2286|0.1995	0.00001	*0.00001*	**0.00014**
129	All_PGEigengap_FiberN	0.0277|0.0236	0.00001	*0.00001*	**0.00016**
463	Left_MinCutBalDivSum_FiberLengthMean	0.0960|0.0810	0.00002	*0.00001*	**0.00023**
83	Left_PGEigengap_FAMean	0.3364|0.3026	0.00001	*0.00001*	**0.00023**
83	Left_MinCutBalDivSum_Unweighted	0.2448|0.2266	0.00001	*0.00001*	**0.00029**
83	All_PGEigengap_FiberN	0.0317|0.0272	0.00001	*0.00001*	**0.00029**
83	All_Sum_FiberNDivLength	471.6398|448.0170	0.00001	*0.00001*	**0.00029**
83	Right_Sum_FiberN	5273.6065|5044.8072	0.00001	*0.00001*	**0.00030**
234	All_Sum_FiberN	13150.0833|12735.7590	0.00001	*0.00001*	**0.00031**
234	Left_MinCutBalDivSum_FiberLengthMean	0.1466|0.1258	0.00001	*0.00001*	**0.00037**
129	Left_MinCutBalDivSum_Unweighted	0.1929|0.1755	0.00001	*0.00001*	**0.00042**
83	Left_Sum_FiberN	5457.7213|5215.3012	0.00001	*0.00001*	**0.00053**
234	Left_PGEigengap_FAMean	0.1445|0.1263	0.00001	*0.00001*	**0.00054**
83	Left_LogSpanningForestN_Unweighted	95.2838|93.4346	0.00117	*0.00001*	**0.00061**
83	All_LogSpanningForestN_FiberN	396.1740|392.13187	0.00001	*0.00001*	**0.00063**
83	Right_Sum_FAMean	103.2289|96.5212	0.00001	*0.00001*	**0.00066**
129	All_Sum_FAMean	388.6029|363.4251	0.00001	*0.00001*	**0.00071**
234	Left_MinCutBalDivSum_Unweighted	0.1402|0.1265	0.00001	*0.00001*	**0.00104**
463	All_Sum_FiberN	13517.1239|13136.1445	0.00001	*0.00001*	**0.00127**
129	Left_LogSpanningForestN_FiberNDivLength	105.4555|102.2015	0.00023	*0.00001*	**0.00150**
234	Left_PGEigengap_FiberLengthMean	0.1495|0.1285	0.00001	*0.00001*	**0.00175**
129	All_LogSpanningForestN_FAMean	191.1538|182.0234	0.00001	*0.00001*	**0.00198**
129	Left_Sum_FiberN	6036.5583|5814.3373	0.00001	*0.00001*	**0.00212**
129	Right_HoffmanBound_FiberNDivLength	2.6708|2.5903	0.00255	*0.00001*	**0.00219**
1015	All_Sum_FiberN	13707.0416|13336.9397	0.00001	*0.00001*	**0.00251**
129	Left_MinCutBalDivSum_FiberLengthMean	0.1986|0.1774	0.00001	*0.00001*	**0.00321**
129	Right_Sum_FAMean	186.3736|174.8309	0.00001	*0.00001*	**0.00332**
234	Right_PGEigengap_FiberNDivLength	0.0702|0.0639	0.00001	*0.00001*	**0.00370**
83	Left_MinCutBalDivSum_FiberLengthMean	0.2368|0.2154	0.00003	*0.00002*	**0.00421**
1015	Left_MinCutBalDivSum_Unweighted	0.0566|0.0497	0.00001	*0.00002*	**0.00455**
83	Right_LogSpanningForestN_FiberNDivLength	69.2438|67.3483	0.00001	*0.00002*	**0.00526**
463	All_MinSpanningForest_FAMean	97.8528|93.9407	0.00001	*0.00002*	**0.00557**
234	All_MinSpanningForest_FAMean	51.1676|48.9654	0.00001	*0.00002*	**0.00572**
129	Right_MinVertexCover_FAMean	14.6370|14.1708	0.00009	*0.00002*	**0.00589**
129	Left_PGEigengap_FiberLengthMean	0.2354|0.2048	0.00001	*0.00003*	**0.00627**
129	Left_Sum_FAMean	192.6335|180.2677	0.00001	*0.00003*	**0.00659**
83	All_MaxMatching_FAMean	18.4793|17.8628	0.00001	*0.00003*	**0.00678**
234	All_Sum_FAMean	674.3861|637.7626	0.00001	*0.00003*	**0.00740**
129	All_MinVertexCover_FAMean	29.2681|28.3448	0.00001	*0.00003*	**0.00769**
129	Right_PGEigengap_Unweighted	0.2018|0.1819	0.00001	*0.00003*	**0.00796**
83	Left_LogSpanningForestN_FiberN	198.7272|196.5478	0.00045	*0.00003*	**0.00813**
129	Right_Sum_FiberN	5865.325|5675.9036	0.00001	*0.00004*	**0.00914**
83	Right_LogSpanningForestN_FAMean	51.1770|48.6093	0.00001	*0.00004*	**0.00915**
129	All_LogSpanningForestN_FiberN	597.6881|592.8658	0.00001	*0.00004*	**0.00918**
129	Right_LogSpanningForestN_FAMean	91.9198|87.7893	0.00001	*0.00004*	**0.00916**
129	Right_PGEigengap_FiberNDivLength	0.0904|525.9763	0.00001	*0.00004*	**0.00925**
1015	Left_MinCutBalDivSum_FiberLengthMean	0.0580|0.0500	0.00197	*0.00004*	**0.00945**
83	Right_Sum_FiberNDivLength	222.8988|213.3314	0.00001	*0.00004*	**0.00973**
83	Left_MinSpanningForest_FiberLengthMean	554.2756|567.6656	0.00146	*0.00004*	**0.00971**
83	All_LogSpanningForestN_Unweighted	191.0620|188.5304	0.00004	*0.00005*	**0.01204**
129	All_MinSpanningForest_FAMean	30.0726|28.5907	0.00001	*0.00006*	**0.01351**
83	Left_Sum_FiberNDivLength	232.2089|221.4498	0.00001	*0.00006*	**0.01385**
129	All_MinSpanningForest_FiberLengthMean	1642.1057|1666.9289	0.00007	*0.00006*	**0.01431**
129	Right_MinSpanningForest_FAMean	15.5645|14.7611	0.00009	*0.00006*	**0.01447**
83	Right_MinSpanningForest_FAMean	10.2995|9.6958	0.00019	*0.00007*	**0.01516**
129	Left_LogSpanningForestN_FAMean	94.4372|89.6353	0.00001	*0.00007*	**0.01539**
129	Left_MinSpanningForest_FiberLengthMean	826.0336|841.6138	0.00016	*0.00007*	**0.01552**

234	Right_MinSpanningForest_FAMean	25.9870|24.8290	0.00008	*0.00007*	**0.01684**
234	Right_MinVertexCover_FAMean	25.6497|24.9170	0.00019	*0.00008*	**0.01794**
83	All_MaxFracMatching_FAMean	18.5112|17.9306	0.00001	*0.00008*	**0.01795**
83	All_MinVertexCover_FAMean	18.5112|17.9306	0.00001	*0.00008*	**0.01787**
129	All_Sum_Unweighted	1008.0166|979.3975	0.00001	*0.00008*	**0.01801**
83	Right_MaxFracMatching_FAMean	9.2021|8.9143	0.00004	*0.00008*	**0.01872**
83	Right_MinVertexCover_FAMean	9.2021|8.9143	0.00004	*0.00008*	**0.01864**
463	Right_MinSpanningForest_FAMean	50.4171|48.29781	0.00005	*0.00009*	**0.01997**
83	Right_PGEigengap_Unweighted	0.2970|0.2748	0.00001	*0.00009*	**0.01995**
234	Left_Sum_FAMean	332.9738|314.1271	0.00001	*0.00010*	**0.02095**
129	Right_PGEigengap_FAMean	0.2242|0.2037	0.00001	*0.00010*	**0.02190**
83	Right_MaxMatching_FAMean	9.1786|8.8951	0.00003	*0.00011*	**0.02264**
83	All_MinSpanningForest_FAMean	19.7614|18.7034	0.00001	*0.00011*	**0.02368**
234	Left_Sum_FiberN	6532.7|6335.9879	0.00001	*0.00012*	**0.02604**
83	All_MinCutBalDivSum_FiberNDivLength	0.03410|0.0289	0.00001	*0.00013*	**0.02719**
83	Left_PGEigengap_FiberLengthMean	0.3342|0.3015	0.00001	*0.00013*	**0.02760**
83	Right_PGEigengap_FiberNDivLength	0.1480|0.1383	0.00001	*0.00014*	**0.02852**
234	Left_MinSpanningForest_FiberLengthMean	1424.5320|1442.5840	0.00028	*0.00015*	**0.03074**
234	All_MinVertexCover_FAMean	51.3348|49.8970	0.00001	*0.00015*	**0.03086**
234	Right_Sum_FAMean	331.7349|314.9959	0.00001	*0.00015*	**0.03164**
83	All_MinSpanningForest_FiberLengthMean	1093.0591|1111.8064	0.00017	*0.00016*	**0.03371**
129	Left_LogSpanningForestN_FiberN	300.1093|297.3925	0.00100	*0.00018*	**0.03788**
129	Right_LogSpanningForestN_FiberNDivLength	100.2820|98.0429	0.00009	*0.00020*	**0.04110**
1015	Left_MinCutBalDivSum_FiberN	0.0479|0.0432	0.00107	*0.00021*	**0.04237**
129	Left_Sum_Unweighted	510.8916|496.0602	0.00001	*0.00022*	**0.04374**
234	Right_PGEigengap_Unweighted	0.1194|0.1079	0.00001	*0.00022*	**0.04430**
129	All_HoffmanBound_Unweighted	4.5868|4.5055	0.00004	*0.00023*	**0.04511**
234	All_Sum_FiberNDivLength	617.1686|598.9007	0.00001	*0.00024*	**0.04709**
129	Right_HoffmanBound_Unweighted	4.6124|4.5159	0.00001	*0.00028*	0.05587
129	Right_MinCutBalDivSum_FiberLengthMean	0.1915|0.1753	0.00001	*0.00035*	0.06880
463	Right_AdjLMaxDivD_Unweighted	1.7949|1.7560	0.00152	*0.00037*	0.07193
1015	Right_AdjLMaxDivD_Unweighted	2.7110|2.6336	0.00147	*0.00037*	0.07307
83	Right_PGEigengap_FAMean	0.3247|0.3030	0.00001	*0.00043*	0.08256
463	Left_Sum_FiberN	6705.2396|6524.7228	0.00001	*0.00043*	0.08268
83	Left_AdjLMaxDivD_FAMean	1.3330|1.3538	0.00276	*0.00043*	0.08304
1015	Right_HoffmanBound_FiberN	2.3184|2.2759	0.00008	*0.00046*	0.08753
129	Right_HoffmanBound_FiberN	2.6444|2.5628	0.00010	*0.00046*	0.08723
234	Right_PGEigengap_FiberN	0.0909|0.0825	0.00001	*0.00048*	0.09081
129	Left_Sum_FiberNDivLength	269.5839|259.7618	0.00014	*0.00048*	0.09059
234	Right_Sum_FiberN	6272.2333|6109.0240	0.00001	*0.00049*	0.09082
234	All_LogSpanningForestN_FiberNDivLength	262.4479|256.2612	0.00295	*0.00049*	0.09065
463	All_Sum_FAMean	1019.7387|973.3478	0.00001	*0.00052*	0.09572
83	Right_MinCutBalDivSum_Unweighted	0.2377|0.2254	0.00001	*0.00058*	0.10656
83	Right_MinCutBalDivSum_FiberNDivLength	0.1280|0.1202	0.00001	*0.00060*	0.11071
463	All_MinCutBalDivSum_FiberN	0.0247|0.0211	0.00001	*0.00062*	0.11205
129	Right_MinCutBalDivSum_Unweighted	0.1884|0.1761	0.00001	*0.00062*	0.11229
1015	Left_Sum_FiberN	6803.8333|6627.2168	0.00006	*0.00068*	0.12183
234	All_MinSpanningForest_FiberLengthMean	2800.7846|2827.1182	0.00013	*0.00069*	0.12438
463	Left_MinSpanningForest_FAMean	47.5061|45.7752	0.00001	*0.00071*	0.12586
83	Right_LogSpanningForestN_FiberN	189.8663|188.1787	0.00001	*0.00072*	0.12740
234	Left_MinSpanningForest_FAMean	25.2698|24.2600	0.00001	*0.00072*	0.12714
463	Left_Sum_FAMean	497.9572|473.8789	0.00001	*0.00078*	0.13569
1015	All_MinCutBalDivSum_FiberN	0.0240|0.0206	0.00001	*0.00080*	0.13954
1015	All_MinSpanningForest_FAMean	201.9819|195.954	0.00010	*0.00088*	0.15161
83	Left_HoffmanBound_Unweighted	4.7029|4.6025	0.00001	*0.00092*	0.15799
234	All_MinCutBalDivSum_FiberN	0.0255|0.0219	0.00001	*0.00093*	0.15874
83	All_HoffmanBound_Unweighted	4.5455|4.4635	0.00001	*0.00095*	0.16097
463	All_Sum_FiberNDivLength	652.6090|636.4074	0.00004	*0.00098*	0.16550
83	Left_AdjLMaxDivD_FiberN	1.9219|1.9959	0.00011	*0.00099*	0.16601
234	Right_HoffmanBound_FiberNDivLength	2.5444|2.492	0.00005	*0.00100*	0.16766
129	Left_MinVertexCover_FAMean	14.4665|14.0373	0.00001	*0.00102*	0.16859
463	Right_HoffmanBound_FiberNDivLength	2.4528|2.4067	0.00005	*0.00113*	0.18633
234	All_LogSpanningForestN_FAMean	326.5297|314.2237	0.00001	*0.00118*	0.19275
234	Right_PGEigengap_FAMean	0.1358|0.1245	0.00001	*0.00123*	0.20085
83	Left_HoffmanBound_FAMean	4.54790|4.4467	0.00004	*0.00124*	0.20121
83	Right_MinCutBalDivSum_FAMean	0.2459|0.2343	0.00001	*0.00127*	0.20401
1015	All_AdjLMaxDivD_Unweighted	2.8024|2.7315	0.00035	*0.00133*	0.21263
129	Right_PGEigengap_FiberLengthMean	0.2294|0.2076	0.00001	*0.00137*	0.21729

234	Left_LogSpanningForestN_FAMean	160.7687|153.7631	0.00001	*0.00149*	0.23541
234	Left_MinVertexCover_FAMean	25.5400|24.8354	0.00001	*0.00155*	0.24260
83	All_MinCutBalDivSum_FiberN	0.0301|0.0262	0.00004	*0.00160*	0.25006
129	All_MinCutBalDivSum_FiberN	0.0270|0.0234	0.00001	*0.00172*	0.26733
463	All_MinCutBalDivSum_FiberNDivLength	0.0247|0.0207	0.00009	*0.00177*	0.27216
83	Left_MaxMatching_FAMean	9.0821|8.8091	0.00001	*0.00182*	0.27878
1015	All_LogSpanningForestN_FiberNDivLength	-350.6301|-371.0974	0.00003	*0.00183*	0.27877
463	Right_Sum_FiberN	6464.768|6321.2048	0.00001	*0.00187*	0.28166
1015	Right_MinSpanningForest_FAMean	104.1024|100.8625	0.00063	*0.00192*	0.28781
463	Left_PGEigengap_FiberN	0.0639|0.0533	0.00077	*0.00193*	0.28752
129	Right_AdjLMaxDivD_Unweighted	1.2663|1.2554	0.00003	*0.00200*	0.29568
234	Right_MinCutBalDivSum_Unweighted	0.1346|0.1248	0.00001	*0.00204*	0.29922
129	Right_PGEigengap_FiberN	0.1213|0.1122	0.00001	*0.00219*	0.31972
1015	Right_HoffmanBound_FiberNDivLength	2.3635|2.3224	0.00001	*0.00223*	0.32298
129	Right_Sum_FiberNDivLength	260.6268|253.0514	0.00002	*0.00224*	0.32299
129	Left_LogSpanningForestN_Unweighted	160.6906|158.8977	0.00181	*0.00230*	0.32843
1015	All_Sum_FiberNDivLength	674.9224|659.8373	0.00051	*0.00242*	0.34341
234	Left_Sum_FiberNDivLength	307.8607|298.9560	0.00180	*0.00251*	0.35422
1015	Left_AdjLMaxDivD_FiberN	7.2625|7.6401	0.00670	*0.00256*	0.35855
1015	Left_LogSpanningForestN_FiberNDivLength	-172.7454|-184.1958	0.00045	*0.00257*	0.35757
1015	Right_Sum_FiberN	6558.825|6419.7951	0.00001	*0.00260*	0.35831
234	Right_MinCutBalDivSum_FiberLengthMean	0.14285|0.1300	0.00001	*0.00261*	0.35696
83	Right_Sum_Unweighted	258.9098|252.433	0.00001	*0.00262*	0.35612
463	Right_Sum_FAMean	511.0996|490.0837	0.00001	*0.00263*	0.35544
129	Left_MinSpanningForest_FAMean	14.6338|13.9911	0.00001	*0.00288*	0.38627
129	Right_HoffmanBound_FAMean	4.4157|4.3338	0.00169	*0.00305*	0.40579
129	All_LogSpanningForestN_Unweighted	320.12464|317.1016	0.00022	*0.00307*	0.40566
83	Left_MaxFracMatching_FAMean	9.1276|8.8673	0.00001	*0.00311*	0.40756
83	Left_MinVertexCover_FAMean	9.1276|8.8673	0.00001	*0.00311*	0.40444
129	Right_Sum_Unweighted	473.3083|461.3614	0.00001	*0.00336*	0.43345
234	Right_LogSpanningForestN_FAMean	161.2133|155.8611	0.00005	*0.00417*	0.53417
83	All_MaxMatching_FiberN	2409.5901|2350.9397	0.00065	*0.00426*	0.54130
1015	Right_MinVertexCover_Unweighted	209.0|212.1686	0.00018	*0.00440*	0.55470
1015	Right_MaxMatching_Unweighted	208.825|211.9036	0.00023	*0.00441*	0.55173
1015	Right_MaxFracMatching_Unweighted	209.0125|212.0963	0.00048	*0.00467*	0.57851
463	Left_AdjLMaxDivD_FiberN	3.9863|4.1652	0.00752	*0.00472*	0.58107
83	All_MaxFracMatching_FiberN	2413.9795|2353.6987	0.00018	*0.00482*	0.58813
83	All_MinVertexCover_FiberN	2413.9795|2353.6987	0.00018	*0.00482*	0.58330
463	Left_PGEigengap_Unweighted	0.0716|0.0609	0.00189	*0.00502*	0.60187
83	Left_MinSpanningForest_FAMean	9.6386|9.1950	0.00001	*0.00509*	0.60605
234	All_Sum_Unweighted	1799.575|1764.3373	0.00001	*0.00521*	0.61534
463	All_MinVertexCover_FAMean	88.9526|86.9509	0.00196	*0.00538*	0.62967
234	Right_PGEigengap_FiberLengthMean	0.1428|0.1302	0.00001	*0.00539*	0.62543
129	Right_LogSpanningForestN_FiberN	289.9664|288.0403	0.00013	*0.00567*	0.65178
83	Right_MinCutBalDivSum_FiberLengthMean	0.2340|0.2218	0.00001	*0.00571*	0.65079
1015	Left_MinSpanningForest_FAMean	98.0028|95.2974	0.00024	*0.00659*	0.74520
463	Left_PGEigengap_FAMean	0.0835|0.07132	0.00319	*0.00661*	0.74045
463	Right_HoffmanBound_FiberN	2.3990|2.36062	0.00045	*0.00671*	0.74462
234	Right_MinCutBalDivSum_FiberN	0.0956|0.0898	0.00001	*0.00702*	0.77239
1015	All_MaxFracMatching_Unweighted	418.3739|424.0783	0.00017	*0.00781*	0.85172
463	Left_PGEigengap_FiberNDivLength	0.0465|0.0403	0.00312	*0.00822*	0.88762
234	Left_Sum_Unweighted	908.525|889.7228	0.00012	*0.00841*	0.89999
463	Left_PGEigengap_FiberLengthMean	0.0880|0.07505	0.00663	*0.00884*	0.93695
1015	Right_MinVertexCoverBinary_Unweighted	228.4|232.75903	0.00179	*0.00898*	0.94273
129	Left_AdjLMaxDivD_FiberN	1.8683|1.9385	0.0006	*0.00905*	0.94119
1015	All_MinVertexCover_Unweighted	417.625|423.2222	0.00009	*0.00924*	0.95151
83	Left_Sum_FiberLengthMean	8672.8595|8232.6217	0.00946	*0.00958*	0.97707
83	Right_PGEigengap_FiberLengthMean	0.3343|0.3134	0.00001	*0.01002*	1.01235
463	Left_MinVertexCover_FAMean	43.4996|42.4814	0.00078	*0.01018*	1.01839
129	Right_MinCutBalDivSum_FiberN	0.1108|0.10464	0.00001	*0.01064*	1.05338
129	Left_HoffmanBound_Unweighted	4.7137|4.6438	0.00040	*0.01072*	1.05098
1015	All_Sum_FAMean	1444.4965|1398.4729	0.00001	*0.01224*	1.18762
463	Right_MinCutBalDivSum_Unweighted	0.0918|0.0861	0.00001	*0.01227*	1.17782
234	All_PGEigengap_FAMean	0.0190|0.0172	0.00376	*0.01229*	1.16788
83	Right_PGEigengap_FiberN	0.1696|0.1605	0.00001	*0.01275*	1.19877
1015	All_MinVertexCoverBinary_Unweighted	457.9416|465.8313	0.00021	*0.01303*	1.21160
129	All_PGEigengap_FAMean	0.0315|0.0288	0.00611	*0.01317*	1.21205
234	Right_HoffmanBound_FiberN	2.5314|2.4861	0.00274	*0.01325*	1.20545

83	All_Sum_FiberLengthMean	16888.8359|16180.3784	0.00227	*0.01364*	1.22751
129	Left_AdjLMaxDivD_FAMean	1.3767|1.3947	0.00366	*0.01387*	1.23455
1015	All_MaxFracMatching_FiberN	2489.2041|2447.0120	0.00001	*0.01440*	1.26697
463	Left_LogSpanningForestN_FAMean	211.7869|202.0911	0.00019	*0.01528*	1.32939
463	All_PGEigengap_FiberNDivLength	0.0177|0.0149	0.0029	*0.01609*	1.38336
1015	Left_AdjLMaxDivD_Unweighted	2.7123|2.6596	0.00095	*0.01668*	1.41741
463	All_AdjLMaxDivD_Unweighted	1.8766|1.8486	0.00023	*0.01701*	1.42876
1015	Left_Sum_FAMean	709.6940|685.9307	0.00003	*0.01734*	1.43905
234	Right_Sum_FiberNDivLength	292.9252|286.7967	0.00046	*0.01734*	1.42191
83	Right_AdjLMaxDivD_Unweighted	1.2546|1.2472	0.00357	*0.01771*	1.43452
1015	All_MaxMatching_FiberN	2486.8166|2446.6626	0.00001	*0.01863*	1.49000
83	Right_HoffmanBound_FiberNDivLength	2.6162|2.5597	0.00012	*0.02098*	1.65710
1015	All_LogSpanningForestN_FAMean	457.4291|429.4160	0.00002	*0.02193*	1.71079
463	Right_MinVertexCoverBinary_Unweighted	138.8842|140.6385	0.00346	*0.02211*	1.70228
463	Right_PGEigengap_FiberNDivLength	0.0510|0.0468	0.00011	*0.02227*	1.69242
463	All_LogSpanningForestN_FAMean	443.0184|426.4406	0.00033	*0.02327*	1.74559
83	All_HoffmanBound_FAMean	4.3274|4.2704	0.00001	*0.02581*	1.90979
234	All_PGEigengap_Unweighted	0.01750|0.0161	0.00442	*0.02653*	1.93685
1015	All_MinVertexCover_FiberN	2491.4166|2452.9036	0.00001	*0.02842*	2.04607
234	All_LogSpanningForestN_FiberN	955.4761|950.29269	0.00178	*0.02972*	2.11030
1015	Right_Sum_FAMean	723.2379|702.4680	0.00005	*0.03022*	2.11561
463	Right_MaxMatching_Unweighted	110.6859|111.2048	0.00204	*0.03132*	2.16131
1015	Right_LogSpanningForestN_FiberNDivLength	-183.9473|-192.2899	0.00128	*0.03399*	2.31135
1015	All_MinCutBalDivSum_Unweighted	0.0066|0.00160	0.00518	*0.03489*	2.33738
1015	Left_LogSpanningForestN_FAMean	217.4574|203.1308	0.00005	*0.03649*	2.40862
129	Right_LogSpanningForestN_Unweighted	153.7504|152.5235	0.00329	*0.03858*	2.50795
1015	Right_LogSpanningForestN_FAMean	233.3453|219.9728	0.00017	*0.03912*	2.50390
234	Right_Sum_Unweighted	866.725|852.1807	0.00005	*0.04054*	2.55409
463	Right_PGEigengap_Unweighted	0.0705|0.0649	0.00008	*0.04133*	2.56240
129	All_PGEigengap_Unweighted	0.0299|0.0279	0.00644	*0.04404*	2.68649
463	Right_MinCutBalDivSum_FiberLengthMean	0.0956|0.0896	0.00001	*0.04584*	2.75064
463	All_MaxFracMatching_Unweighted	222.3057|223.2168	0.00011	*0.04727*	2.78883
463	All_MaxMatching_Unweighted	222.1735|223.0120	0.00006	0.05127	2.97341
83	All_MaxFracMatching_FiberNDivLength	109.988|107.7572	0.00221	0.05169	2.94617
83	All_MinVertexCover_FiberNDivLength	109.9887|107.7572	0.00221	0.05169	2.89448
463	All_MinVertexCover_Unweighted	222.3388|223.2289	0.00010	0.05250	2.88729
463	Right_Sum_FiberNDivLength	311.3203|306.3797	0.00331	0.05424	2.92881
463	Right_PGEigengap_FiberN	0.0661|0.0611	0.00009	0.05453	2.89026
463	Right_MinVertexCover_Unweighted	110.9380|111.4216	0.00259	0.05539	2.88018
1015	Left_MinVertexCoverBinary_Unweighted	229.2583|232.6746	0.00034	0.05953	3.03594
1015	Left_MaxFracMatching_Unweighted	209.3666|211.7048	0.00118	0.06019	3.00930
463	Right_MaxFracMatching_Unweighted	110.9256|111.3855	0.00309	0.06695	3.28031
129	All_Sum_FiberLengthMean	30088.0733|29148.1015	0.00412	0.07048	3.38324
83	Right_LogSpanningForestN_Unweighted	89.7645|89.0820	0.00089	0.07272	3.41764
1015	Left_MinVertexCover_Unweighted	209.1916|211.4337	0.00104	0.07578	3.48604
1015	Right_MinSpanningForest_FiberN	473.825|477.9277	0.00337	0.07800	3.51010
463	All_MinVertexCoverBinary_Unweighted	277.2396|279.67469	0.00020	0.07991	3.51606
129	All_HoffmanBound_FAMean	4.3668|4.3276	0.00372	0.08437	3.62771
234	Right_MinSpanningForest_FiberLengthMean	1370.9407|1378.45522	0.00707	0.08438	3.54390
463	All_MinSpanningForest_FiberLengthMean	5356.4309|5385.0509	0.00627	0.08536	3.49962
83	Right_HoffmanBound_Unweighted	4.6113|4.5608	0.00006	0.08584	3.43357
463	Right_LogSpanningForestN_FAMean	225.9686|219.2312	0.00395	0.08786	3.42655
463	Right_PGEigengap_FAMean	0.0826|0.0771	0.00007	0.08842	3.35998
1015	Left_MaxMatching_Unweighted	209.2833|211.36143	0.00055	0.08938	3.30714
83	All_MaxMatching_FiberNDivLength	109.5831|107.6791	0.00650	0.09423	3.39233
83	Right_MinCutBalDivSum_FiberN	0.1287|0.12476	0.00001	0.09707	3.39755
129	Right_Sum_FiberLengthMean	13908.4549|13470.3465	0.00837	0.10380	3.52934
83	Right_HoffmanBound_FAMean	4.4308|4.3780	0.00001	0.10389	3.42821
83	Right_Sum_FiberLengthMean	7631.6042|7392.1456	0.00300	0.11548	3.69535
463	Left_MaxFracMatching_Unweighted	111.3760|111.8072	0.00022	0.13062	4.04908
463	Right_PGEigengap_FiberLengthMean	0.0857|0.08013	0.00052	0.13259	3.97768
463	All_AdjLMaxDivD_FiberNDivLength	5.0105|4.8826	0.00063	0.14493	4.20303
234	All_Sum_FiberLengthMean	50705.6991|49509.7827	0.00472	0.14614	4.09197
1015	All_MinSpanningForest_FiberN	951.7333|956.9518	0.00215	0.14956	4.03800
129	All_AdjLMaxDivD_Unweighted	1.3009|1.2950	0.00391	0.15217	3.95652
463	All_MaxMatching_FiberN	2408.5619|2385.506	0.00003	0.16612	4.15299
234	Left_Sum_FiberLengthMean	25968.3400|25333.5608	0.00808	0.16909	4.05805
463	Left_MinSpanningForest_FiberLengthMean	2693.9037|2708.0471	0.00029	0.16935	3.89511

463	Left_MinVertexCover_Unweighted	111.3966|111.7831	0.00020	0.17471	3.84373
463	Right_MinCutBalDivSum_FiberN	0.0720|0.0698	0.00001	0.18380	3.85985
1015	Right_MinCutBalDivSum_Unweighted	0.0567|0.0548	0.00001	0.20002	4.00031
463	All_MinVertexCover_FiberN	2414.3842|2392.3313	0.00003	0.20040	3.80760
463	Left_AdjLMaxDivD_Unweighted	1.8456|1.8312	0.00037	0.21019	3.78341
463	All_MaxFracMatching_FiberN	2414.38429|2393.3915	0.00004	0.22266	3.78530
1015	All_AdjLMaxDivD_FiberNDivLength	9.8369|9.6047	0.00022	0.23128	3.70054
234	All_AdjLMaxDivD_FiberNDivLength	2.9784|2.9251	0.00578	0.23787	3.56812
234	Right_LogSpanningForestN_FiberN	464.4918|462.8764	0.00579	0.23915	3.34810
1015	Right_MinSpanningForest_FiberNDivLength	21.3594|21.58465	0.00392	0.33018	4.29233
463	Left_MaxMatching_Unweighted	111.2975|111.5662	0.00011	0.33520	4.02238
234	All_MaxMatching_FiberN	2403.8|2387.5180	0.00037	0.33724	3.70960
129	All_MaxFracMatching_FiberN	2418.9208|2403.0180	0.00167	0.34902	3.49021
1015	Right_MinCutBalDivSum_FiberLengthMean	0.0577|0.0559	0.00006	0.35609	3.20477
129	All_MaxMatching_FiberN	2412.5|2396.9156	0.00155	0.36285	2.90276
463	Left_MinVertexCoverBinary_Unweighted	138.0165|138.6987	0.00028	0.37344	2.61406
129	All_MinVertexCover_FiberN	2415.5041|2401.6325	0.00145	0.43115	2.58690
1015	Right_MinCutBalDivSum_FiberN	0.0470|0.0462	0.00001	0.45084	2.25419
234	All_MinVertexCover_FiberN	2411.820|2399.3012	0.00010	0.46896	1.87582
234	All_MaxFracMatching_FiberN	2409.7791|2398.1445	0.00032	0.49808	1.49424
129	Left_AdjLMaxDivD_Unweighted	1.2641|1.2625	0.00166	0.62457	1.24914
1015	All_MinSpanningForest_FiberNDivLength	42.7199|42.8768	0.00901	0.66464	0.66464

Table S2

In this table, we give the graph-theoretic parameters computed for the 83-vertex graphs. The table contains their arithmetic means in the male and female groups, and the corresponding p-values for group 0 (see the "Statistical analysis" subsection). The graph-parameters and the syntax of the data are defined in the main text. Significant differences ($p < 0.01$) are denoted with an asterisk in the last column.

Property	Female	Male	p-value	
All_AdjLMaxDivD_FAMean	1.36875	1.38259	0.01187	
All_AdjLMaxDivD_FiberLengthMean	1.44867	1.44343	0.54869	
All_AdjLMaxDivD_FiberN	2.05880	2.11497	0.01035	
All_AdjLMaxDivD_FiberNDivLength	1.86494	1.86145	0.80141	
All_AdjLMaxDivD_Unweighted	1.26969	1.26372	0.07134	
All_HoffmanBound_FAMean	4.33196	4.19675	0.00001	*
All_HoffmanBound_FiberLengthMean	3.22244	3.18444	0.15967	
All_HoffmanBound_FiberN	2.62571	2.59760	0.14184	
All_HoffmanBound_FiberNDivLength	2.50583	2.46965	0.06702	
All_HoffmanBound_Unweighted	4.54119	4.39769	0.00001	*
All_LeftRatio_FAMean	0.96407	0.96324	0.87951	
All_LeftRatio_FiberLengthMean	1.01790	1.02383	0.40343	
All_LeftRatio_FiberN	0.98798	0.99695	0.09018	
All_LeftRatio_FiberNDivLength	0.98714	0.99320	0.21746	
All_LeftRatio_Unweighted	0.99363	0.99763	0.29970	
All_LogSpanningForestN_FAMean	108.57625	99.82325	0.00001	*
All_LogSpanningForestN_FiberLengthMean	454.30207	452.11354	0.15516	
All_LogSpanningForestN_FiberN	395.98463	391.08233	0.00001	*
All_LogSpanningForestN_FiberNDivLength	147.77314	142.42711	0.00001	*
All_LogSpanningForestN_Unweighted	190.48531	187.73945	0.00004	*
All_MaxFracMatching_FAMean	18.46840	17.58077	0.00001	*
All_MaxFracMatching_FiberLengthMean	2009.76723	1975.53559	0.34678	
All_MaxFracMatching_FiberN	2426.47391	2344.52660	0.00018	*

All_MaxFracMatching_FiberNDivLength	111.31000	107.40786	0.00221	*
All_MaxFracMatching_Unweighted	40.80000	40.94681	0.01166	
All_MaxMatching_FAMean	18.44825	17.52991	0.00001	*
All_MaxMatching_FiberLengthMean	2008.89813	1979.58130	0.41489	
All_MaxMatching_FiberN	2418.51304	2346.44681	0.00065	*
All_MaxMatching_FiberNDivLength	110.89315	107.45589	0.00650	*
All_MaxMatching_Unweighted	40.53043	40.67021	0.04499	
All_MinCutBalDivSum_FAMean	0.04102	0.03885	0.12673	
All_MinCutBalDivSum_FiberLengthMean	0.03239	0.03182	0.74907	
All_MinCutBalDivSum_FiberN	0.02940	0.02436	0.00004	*
All_MinCutBalDivSum_FiberNDivLength	0.03340	0.02661	0.00001	*
All_MinCutBalDivSum_Unweighted	0.04008	0.03789	0.11561	
All_MinSpanningForest_FAMean	19.67830	18.19979	0.00001	*
All_MinSpanningForest_FiberLengthMean	1093.76872	1112.08242	0.00017	*
All_MinSpanningForest_FiberN	101.08696	103.47872	0.03254	
All_MinSpanningForest_FiberNDivLength	3.64724	3.70903	0.45315	
All_MinVertexCoverBinary_Unweighted	59.19130	59.07447	0.52716	
All_MinVertexCover_FAMean	18.46840	17.58077	0.00001	*
All_MinVertexCover_FiberLengthMean	2009.76723	1975.53559	0.34678	
All_MinVertexCover_FiberN	2426.47391	2344.52660	0.00018	*
All_MinVertexCover_FiberNDivLength	111.31000	107.40786	0.00221	*
All_MinVertexCover_Unweighted	40.80000	40.94681	0.01166	
All_PGEigengap_FAMean	0.05398	0.05032	0.03291	
All_PGEigengap_FiberLengthMean	0.04269	0.04155	0.58068	
All_PGEigengap_FiberN	0.03111	0.02622	0.00001	*
All_PGEigengap_FiberNDivLength	0.03400	0.02787	0.00001	*
All_PGEigengap_Unweighted	0.05187	0.04818	0.01977	
All_Sum_FAMean	217.42826	195.09313	0.00001	*
All_Sum_FiberLengthMean	16574.03831	15653.05783	0.00227	*
All_Sum_FiberN	11129.93913	10353.87234	0.00001	*
All_Sum_FiberNDivLength	475.82094	445.36396	0.00001	*
All_Sum_Unweighted	560.69565	537.21277	0.00001	*
Left_AdjLMaxDivD_FAMean	1.33861	1.35695	0.00276	*
Left_AdjLMaxDivD_FiberLengthMean	1.39587	1.38727	0.25905	
Left_AdjLMaxDivD_FiberN	1.93777	2.02636	0.00011	*
Left_AdjLMaxDivD_FiberNDivLength	1.73693	1.77462	0.01256	
Left_AdjLMaxDivD_Unweighted	1.24166	1.23423	0.02696	
Left_HoffmanBound_FAMean	4.53589	4.39998	0.00004	*
Left_HoffmanBound_FiberLengthMean	3.26475	3.21357	0.09120	
Left_HoffmanBound_FiberN	2.71677	2.69771	0.41879	
Left_HoffmanBound_FiberNDivLength	2.65881	2.61979	0.16775	
Left_HoffmanBound_Unweighted	4.69319	4.53245	0.00001	*
Left_LogSpanningForestN_FAMean	52.60202	47.79692	0.00001	*
Left_LogSpanningForestN_FiberLengthMean	228.00986	227.07923	0.30936	
Left_LogSpanningForestN_FiberN	198.46664	196.34346	0.00045	*
Left_LogSpanningForestN_FiberNDivLength	73.79704	71.25881	0.00001	*
Left_LogSpanningForestN_Unweighted	94.79835	93.39721	0.00117	*
Left_MaxFracMatching_FAMean	9.13784	8.64312	0.00001	*
Left_MaxFracMatching_FiberLengthMean	1055.51350	1041.51094	0.50266	
Left_MaxFracMatching_FiberN	1168.49565	1155.44681	0.33894	

Left_MaxFracMatching_FiberNDivLength	54.85027	53.06283	0.01912	
Left_MaxFracMatching_Unweighted	20.70870	20.78723	0.03546	
Left_MaxMatching_FAMean	9.11166	8.60492	0.00001	*
Left_MaxMatching_FiberLengthMean	1052.74695	1046.89693	0.77946	
Left_MaxMatching_FiberN	1165.86087	1157.89362	0.54454	
Left_MaxMatching_FiberNDivLength	54.71769	53.09956	0.02948	
Left_MaxMatching_Unweighted	20.44348	20.59574	0.02850	
Left_MinCutBalDivSum_FAMean	0.24446	0.22566	0.00001	*
Left_MinCutBalDivSum_FiberLengthMean	0.23339	0.21318	0.00003	*
Left_MinCutBalDivSum_FiberN	0.12873	0.11341	0.00001	*
Left_MinCutBalDivSum_FiberNDivLength	0.13168	0.11676	0.00001	*
Left_MinCutBalDivSum_Unweighted	0.24046	0.22316	0.00001	*
Left_MinSpanningForest_FAMean	9.64023	8.84224	0.00001	*
Left_MinSpanningForest_FiberLengthMean	555.69180	565.67296	0.00146	*
Left_MinSpanningForest_FiberN	52.26087	54.69149	0.02852	
Left_MinSpanningForest_FiberNDivLength	1.85125	1.95886	0.15079	
Left_MinVertexCoverBinary_Unweighted	29.99130	29.89362	0.50013	
Left_MinVertexCover_FAMean	9.13784	8.64312	0.00001	*
Left_MinVertexCover_FiberLengthMean	1055.51350	1041.51094	0.50266	
Left_MinVertexCover_FiberN	1168.49565	1155.44681	0.33894	
Left_MinVertexCover_FiberNDivLength	54.85027	53.06283	0.01912	
Left_MinVertexCover_Unweighted	20.70870	20.78723	0.03546	
Left_PGEigengap_FAMean	0.32901	0.29424	0.00001	*
Left_PGEigengap_FiberLengthMean	0.32651	0.29063	0.00001	*
Left_PGEigengap_FiberN	0.16362	0.14119	0.00001	*
Left_PGEigengap_FiberNDivLength	0.13964	0.12469	0.00001	*
Left_PGEigengap_Unweighted	0.30169	0.26858	0.00001	*
Left_Sum_FAMean	104.90524	93.93956	0.00001	*
Left_Sum_FiberLengthMean	8454.81314	8010.01349	0.00946	*
Left_Sum_FiberN	5496.91304	5155.82979	0.00001	*
Left_Sum_FiberNDivLength	234.67696	221.00148	0.00001	*
Left_Sum_Unweighted	278.78261	267.84043	0.00001	*
Right_AdjLMaxDivD_FAMean	1.34243	1.34454	0.71444	
Right_AdjLMaxDivD_FiberLengthMean	1.41086	1.41093	0.99277	
Right_AdjLMaxDivD_FiberN	2.05169	2.10053	0.03725	
Right_AdjLMaxDivD_FiberNDivLength	1.79625	1.79740	0.93925	
Right_AdjLMaxDivD_Unweighted	1.25612	1.24659	0.00357	*
Right_HoffmanBound_FAMean	4.46780	4.30965	0.00001	*
Right_HoffmanBound_FiberLengthMean	3.34214	3.31686	0.41097	
Right_HoffmanBound_FiberN	2.64848	2.59375	0.01381	
Right_HoffmanBound_FiberNDivLength	2.62271	2.52700	0.00012	*
Right_HoffmanBound_Unweighted	4.61029	4.48940	0.00006	*
Right_LogSpanningForestN_FAMean	50.77679	47.15788	0.00001	*
Right_LogSpanningForestN_FiberLengthMean	217.22296	216.02568	0.17484	
Right_LogSpanningForestN_FiberN	189.95076	187.56201	0.00001	*
Right_LogSpanningForestN_FiberNDivLength	69.45578	67.16406	0.00001	*
Right_LogSpanningForestN_Unweighted	89.59331	88.39743	0.00089	*
Right_MaxFracMatching_FAMean	9.15030	8.78620	0.00004	*
Right_MaxFracMatching_FiberLengthMean	934.52521	912.03714	0.22786	
Right_MaxFracMatching_FiberN	1169.82174	1154.66489	0.25487	

Right_MaxFracMatching_FiberNDivLength	54.37426	53.80631	0.44887	
Right_MaxFracMatching_Unweighted	20.09130	20.17021	0.10789	
Right_MaxMatching_FAMean	9.12190	8.74960	0.00003	*
Right_MaxMatching_FiberLengthMean	935.16155	909.69912	0.16516	
Right_MaxMatching_FiberN	1167.60870	1154.00000	0.29447	
Right_MaxMatching_FiberNDivLength	54.16573	53.82448	0.64463	
Right_MaxMatching_Unweighted	19.83478	19.89362	0.22290	
Right_MinCutBalDivSum_FAMean	0.24426	0.22199	0.00001	*
Right_MinCutBalDivSum_FiberLengthMean	0.23356	0.21038	0.00001	*
Right_MinCutBalDivSum_FiberN	0.13295	0.11836	0.00001	*
Right_MinCutBalDivSum_FiberNDivLength	0.12923	0.11715	0.00001	*
Right_MinCutBalDivSum_Unweighted	0.23636	0.21557	0.00001	*
Right_MinSpanningForest_FAMean	10.18720	9.53901	0.00019	*
Right_MinSpanningForest_FiberLengthMean	535.98644	542.59066	0.02855	
Right_MinSpanningForest_FiberN	51.97391	52.17021	0.80170	
Right_MinSpanningForest_FiberNDivLength	1.95546	1.95190	0.94388	
Right_MinVertexCoverBinary_Unweighted	28.72174	28.82979	0.38707	
Right_MinVertexCover_FAMean	9.15030	8.78620	0.00004	*
Right_MinVertexCover_FiberLengthMean	934.52521	912.03714	0.22786	
Right_MinVertexCover_FiberN	1169.82174	1154.66489	0.25487	
Right_MinVertexCover_FiberNDivLength	54.37426	53.80631	0.44887	
Right_MinVertexCover_Unweighted	20.09130	20.17021	0.10789	
Right_PGEigengap_FAMean	0.32003	0.28361	0.00001	*
Right_PGEigengap_FiberLengthMean	0.32906	0.28911	0.00001	*
Right_PGEigengap_FiberN	0.17370	0.15054	0.00001	*
Right_PGEigengap_FiberNDivLength	0.14931	0.13170	0.00001	*
Right_PGEigengap_Unweighted	0.29208	0.25911	0.00001	*
Right_Sum_FAMean	102.93685	92.99858	0.00001	*
Right_Sum_FiberLengthMean	7545.43741	7105.85395	0.00300	*
Right_Sum_FiberN	5297.13913	4936.30851	0.00001	*
Right_Sum_FiberNDivLength	224.73904	212.15506	0.00001	*
Right_Sum_Unweighted	258.13043	247.79787	0.00001	*

Table S3

In this table, we give the graph-theoretic parameters computed for the 129-vertex graphs. The table contains their arithmetic means in the male and female groups, and the corresponding p-values for group 0 (see the "Statistical analysis" subsection). The graph-parameters and the syntax of the data are defined in the main text. Significant differences ($p < 0.01$) are denoted with an asterisk in the last column.

Property	Female	Male	p-value	
All_AdjLMaxDivD_FAMean	1.41064	1.42178	0.08520	
All_AdjLMaxDivD_FiberLengthMean	1.49498	1.49885	0.68478	
All_AdjLMaxDivD_FiberN	2.18019	2.21788	0.12879	
All_AdjLMaxDivD_FiberNDivLength	2.09142	2.04274	0.02144	
All_AdjLMaxDivD_Unweighted	1.30068	1.28949	0.00391	*
All_HoffmanBound_FAMean	4.37021	4.30605	0.00372	*
All_HoffmanBound_FiberLengthMean	3.23335	3.22805	0.84136	

All_HoffmanBound_FiberN	2.50847	2.50227	0.73403	
All_HoffmanBound_FiberNDivLength	2.38060	2.42236	0.04465	
All_HoffmanBound_Unweighted	4.59120	4.49569	0.00004	*
All_LeftRatio_FAMean	0.99194	0.99322	0.80077	
All_LeftRatio_FiberLengthMean	1.03504	1.03831	0.59672	
All_LeftRatio_FiberN	0.98611	0.99449	0.08462	
All_LeftRatio_FiberNDivLength	0.98580	0.99159	0.20592	
All_LeftRatio_Unweighted	1.01296	1.01593	0.37175	
All_LogSpanningForestN_FAMean	190.78404	178.44384	0.00001	*
All_LogSpanningForestN_FiberLengthMean	734.96618	731.41772	0.13256	
All_LogSpanningForestN_FiberN	597.06042	591.32147	0.00001	*
All_LogSpanningForestN_FiberNDivLength	210.12907	204.62471	0.00001	*
All_LogSpanningForestN_Unweighted	319.83789	316.08121	0.00022	*
All_MaxFracMatching_FAMean	49.65353	50.66528	0.66683	
All_MaxFracMatching_FiberLengthMean	3200.56758	3155.33612	0.40310	
All_MaxFracMatching_FiberN	2439.32018	2379.91489	0.00167	*
All_MaxFracMatching_FiberNDivLength	129.54868	128.84984	0.66147	
All_MaxFracMatching_Unweighted	63.77193	63.92021	0.01429	
All_MaxMatching_FAMean	49.46172	50.45366	0.67164	
All_MaxMatching_FiberLengthMean	3195.95938	3150.79561	0.40248	
All_MaxMatching_FiberN	2433.70175	2373.79787	0.00155	*
All_MaxMatching_FiberNDivLength	129.14480	128.43086	0.65416	
All_MaxMatching_Unweighted	63.51754	63.63830	0.09249	
All_MinCutBalDivSum_FAMean	0.04566	0.04964	0.19728	
All_MinCutBalDivSum_FiberLengthMean	0.01816	0.01776	0.69091	
All_MinCutBalDivSum_FiberN	0.02662	0.02160	0.00001	*
All_MinCutBalDivSum_FiberNDivLength	0.04857	0.04496	0.15218	
All_MinCutBalDivSum_Unweighted	0.02263	0.02118	0.06676	
All_MinSpanningForest_FAMean	29.88114	27.89533	0.00001	*
All_MinSpanningForest_FiberLengthMean	1638.10279	1661.92149	0.00007	*
All_MinSpanningForest_FiberN	140.42105	141.07447	0.38283	
All_MinSpanningForest_FiberNDivLength	4.49632	4.44670	0.56203	
All_MinVertexCoverBinary_Unweighted	95.98246	95.95745	0.90908	
All_MinVertexCover_FAMean	29.21538	27.95197	0.00001	*
All_MinVertexCover_FiberLengthMean	3199.42585	3154.86947	0.40926	
All_MinVertexCover_FiberN	2440.16228	2379.91489	0.00145	*
All_MinVertexCover_FiberNDivLength	121.15928	119.62335	0.21405	
All_MinVertexCover_Unweighted	63.76754	63.92021	0.01118	
All_PGEigengap_FAMean	0.03177	0.02898	0.00611	*
All_PGEigengap_FiberLengthMean	0.02543	0.02424	0.33973	
All_PGEigengap_FiberN	0.02737	0.02259	0.00001	*
All_PGEigengap_FiberNDivLength	0.02816	0.02267	0.00001	*
All_PGEigengap_Unweighted	0.03039	0.02780	0.00644	*
All_Sum_FAMean	388.40124	352.11586	0.00001	*
All_Sum_FiberLengthMean	29828.05826	28284.32274	0.00412	*
All_Sum_FiberN	12257.67544	11610.31915	0.00001	*
All_Sum_FiberNDivLength	549.08397	524.10766	0.00001	*
All_Sum_Unweighted	1006.11404	966.30851	0.00001	*
Left_AdjLMaxDivD_FAMean	1.38198	1.40166	0.00366	*
Left_AdjLMaxDivD_FiberLengthMean	1.42577	1.42392	0.81274	

Left_AdjLMaxDivD_FiberN	1.87505	1.96425	0.00064	*
Left_AdjLMaxDivD_FiberNDivLength	1.78286	1.82451	0.03311	
Left_AdjLMaxDivD_Unweighted	1.26570	1.25571	0.00166	*
Left_HoffmanBound_FAMean	4.54921	4.48547	0.03131	
Left_HoffmanBound_FiberLengthMean	3.27052	3.26083	0.73075	
Left_HoffmanBound_FiberN	2.75397	2.75802	0.85517	
Left_HoffmanBound_FiberNDivLength	2.66995	2.66242	0.73338	
Left_HoffmanBound_Unweighted	4.73359	4.63754	0.00040	*
Left_LogSpanningForestN_FAMean	94.49928	87.83720	0.00001	*
Left_LogSpanningForestN_FiberLengthMean	369.71301	368.14978	0.22674	
Left_LogSpanningForestN_FiberN	299.76741	297.25592	0.00100	*
Left_LogSpanningForestN_FiberNDivLength	105.37888	102.73539	0.00023	*
Left_LogSpanningForestN_Unweighted	160.53197	158.68211	0.00181	*
Left_MaxFracMatching_FAMean	24.76152	25.29477	0.66522	
Left_MaxFracMatching_FiberLengthMean	1663.09920	1643.90725	0.52787	
Left_MaxFracMatching_FiberN	1167.39912	1152.96809	0.23422	
Left_MaxFracMatching_FiberNDivLength	63.38229	62.99647	0.67411	
Left_MaxFracMatching_Unweighted	32.19298	32.26064	0.07928	
Left_MaxMatching_FAMean	24.57217	25.09906	0.66544	
Left_MaxMatching_FiberLengthMean	1660.02044	1641.59413	0.54314	
Left_MaxMatching_FiberN	1164.21053	1150.29787	0.25202	
Left_MaxMatching_FiberNDivLength	63.13867	62.73563	0.65871	
Left_MaxMatching_Unweighted	31.97368	31.96809	0.81135	
Left_MinCutBalDivSum_FAMean	0.39150	0.41499	0.30927	
Left_MinCutBalDivSum_FiberLengthMean	0.19430	0.17341	0.00001	*
Left_MinCutBalDivSum_FiberN	0.12108	0.10120	0.00001	*
Left_MinCutBalDivSum_FiberNDivLength	0.29168	0.29222	0.97721	
Left_MinCutBalDivSum_Unweighted	0.19064	0.17241	0.00001	*
Left_MinSpanningForest_FAMean	14.54348	13.51233	0.00001	*
Left_MinSpanningForest_FiberLengthMean	824.76247	838.66139	0.00016	*
Left_MinSpanningForest_FiberN	71.20175	72.77660	0.11052	
Left_MinSpanningForest_FiberNDivLength	2.27718	2.29001	0.85866	
Left_MinVertexCoverBinary_Unweighted	48.64035	48.59574	0.77023	
Left_MinVertexCover_FAMean	14.51869	13.77702	0.00001	*
Left_MinVertexCover_FiberLengthMean	1663.75423	1643.77929	0.51100	
Left_MinVertexCover_FiberN	1167.29825	1152.96809	0.23644	
Left_MinVertexCover_FiberNDivLength	59.17693	58.52190	0.38417	
Left_MinVertexCover_Unweighted	32.18860	32.26064	0.06131	
Left_PGEigengap_FAMean	0.22060	0.19382	0.00001	*
Left_PGEigengap_FiberLengthMean	0.22653	0.19611	0.00001	*
Left_PGEigengap_FiberN	0.11793	0.09781	0.00001	*
Left_PGEigengap_FiberNDivLength	0.09286	0.07972	0.00001	*
Left_PGEigengap_Unweighted	0.19796	0.17162	0.00001	*
Left_Sum_FAMean	192.60222	174.73153	0.00001	*
Left_Sum_FiberLengthMean	15444.91099	14683.45752	0.01028	
Left_Sum_FiberN	6041.00877	5772.81915	0.00001	*
Left_Sum_FiberNDivLength	270.61012	259.95554	0.00014	*
Left_Sum_Unweighted	509.72807	490.96809	0.00001	*
Right_AdjLMaxDivD_FAMean	1.37358	1.37371	0.98425	
Right_AdjLMaxDivD_FiberLengthMean	1.43491	1.44388	0.32229	

Right_AdjLMaxDivD_FiberN	2.13247	2.19242	0.04382	
Right_AdjLMaxDivD_FiberNDivLength	1.86174	1.85384	0.68280	
Right_AdjLMaxDivD_Unweighted	1.26948	1.25386	0.00003	*
Right_HoffmanBound_FAMean	4.42153	4.33327	0.00169	*
Right_HoffmanBound_FiberLengthMean	3.32244	3.31805	0.87546	
Right_HoffmanBound_FiberN	2.65165	2.56198	0.00010	*
Right_HoffmanBound_FiberNDivLength	2.66235	2.60715	0.00255	*
Right_HoffmanBound_Unweighted	4.61996	4.50591	0.00001	*
Right_LogSpanningForestN_FAMean	91.35003	85.96894	0.00001	*
Right_LogSpanningForestN_FiberLengthMean	356.43808	354.61854	0.17398	
Right_LogSpanningForestN_FiberN	289.75572	286.91867	0.00013	*
Right_LogSpanningForestN_FiberNDivLength	100.25279	97.89746	0.00009	*
Right_LogSpanningForestN_Unweighted	153.49975	151.79142	0.00329	*
Right_MaxFracMatching_FAMean	24.77565	25.22270	0.69622	
Right_MaxFracMatching_FiberLengthMean	1520.51801	1494.98889	0.35418	
Right_MaxFracMatching_FiberN	1186.44298	1192.61170	0.62145	
Right_MaxFracMatching_FiberNDivLength	64.15821	65.33536	0.19327	
Right_MaxFracMatching_Unweighted	31.58333	31.66489	0.10839	
Right_MaxMatching_FAMean	24.59989	25.05318	0.68852	
Right_MaxMatching_FiberLengthMean	1517.57112	1492.71983	0.36588	
Right_MaxMatching_FiberN	1184.06140	1188.95745	0.69571	
Right_MaxMatching_FiberNDivLength	63.89435	64.98231	0.22657	
Right_MaxMatching_Unweighted	31.33333	31.44681	0.11506	
Right_MinCutBalDivSum_FAMean	0.37589	0.38949	0.51341	
Right_MinCutBalDivSum_FiberLengthMean	0.19107	0.16602	0.00001	*
Right_MinCutBalDivSum_FiberN	0.11322	0.09749	0.00001	*
Right_MinCutBalDivSum_FiberNDivLength	0.27941	0.27422	0.76733	
Right_MinCutBalDivSum_Unweighted	0.18819	0.16752	0.00001	*
Right_MinSpanningForest_FAMean	15.44134	14.52785	0.00009	*
Right_MinSpanningForest_FiberLengthMean	809.70438	818.75455	0.01354	
Right_MinSpanningForest_FiberN	70.29825	69.92553	0.47065	
Right_MinSpanningForest_FiberNDivLength	2.33507	2.28667	0.32437	
Right_MinVertexCoverBinary_Unweighted	46.94737	47.04255	0.54948	
Right_MinVertexCover_FAMean	14.53416	14.02524	0.00009	*
Right_MinVertexCover_FiberLengthMean	1518.88778	1494.74150	0.38002	
Right_MinVertexCover_FiberN	1186.40351	1192.61170	0.62123	
Right_MinVertexCover_FiberNDivLength	59.96806	60.58959	0.42947	
Right_MinVertexCover_Unweighted	31.58333	31.66489	0.10839	
Right_PGEigengap_FAMean	0.22308	0.19156	0.00001	*
Right_PGEigengap_FiberLengthMean	0.22862	0.19449	0.00001	*
Right_PGEigengap_FiberN	0.12475	0.10411	0.00001	*
Right_PGEigengap_FiberNDivLength	0.10095	0.08711	0.00001	*
Right_PGEigengap_Unweighted	0.20057	0.17212	0.00001	*
Right_Sum_FAMean	186.05021	169.14759	0.00001	*
Right_Sum_FiberLengthMean	13793.60649	13074.49495	0.00837	*
Right_Sum_FiberN	5876.42105	5578.19149	0.00001	*
Right_Sum_FiberNDivLength	262.24767	252.08253	0.00002	*
Right_Sum_Unweighted	472.35965	454.00000	0.00001	*

Table S4

In this table, we give the graph-theoretic parameters computed for the 234-vertex graphs. The table contains their arithmetic means in the male and female groups, and the corresponding p-values for group 0 (see the "Statistical analysis" subsection). The graph-parameters and the syntax of the data are defined in the main text. Significant differences ($p < 0.01$) are denoted with an asterisk in the last column.

Property	Female	Male	p-value	
All_AdjLMaxDivD_FAMean	1.60508	1.61546	0.23293	
All_AdjLMaxDivD_FiberLengthMean	1.72307	1.73537	0.35606	
All_AdjLMaxDivD_FiberN	3.03189	3.02754	0.90851	
All_AdjLMaxDivD_FiberNDivLength	3.02286	2.90195	0.00578	*
All_AdjLMaxDivD_Unweighted	1.44658	1.43449	0.03742	
All_HoffmanBound_FAMean	4.06651	4.07749	0.59508	
All_HoffmanBound_FiberLengthMean	3.10629	3.14528	0.12360	
All_HoffmanBound_FiberN	2.34706	2.37219	0.13150	
All_HoffmanBound_FiberNDivLength	2.27630	2.30274	0.10670	
All_HoffmanBound_Unweighted	4.23289	4.21785	0.45061	
All_LeftRatio_FAMean	0.98930	0.98854	0.87192	
All_LeftRatio_FiberLengthMean	1.02634	1.02701	0.91245	
All_LeftRatio_FiberN	0.99363	1.00228	0.06766	
All_LeftRatio_FiberNDivLength	0.99635	1.00247	0.17117	
All_LeftRatio_Unweighted	1.01073	1.01336	0.46683	
All_LogSpanningForestN_FAMean	325.93700	306.72147	0.00001	*
All_LogSpanningForestN_FiberLengthMean	1310.63404	1303.74187	0.10907	
All_LogSpanningForestN_FiberN	953.27020	946.29487	0.00178	*
All_LogSpanningForestN_FiberNDivLength	260.96946	255.95413	0.00295	*
All_LogSpanningForestN_Unweighted	570.62920	565.61983	0.01393	
All_MaxFracMatching_FAMean	80.76738	82.78486	0.65868	
All_MaxFracMatching_FiberLengthMean	5173.35435	5071.70659	0.22429	
All_MaxFracMatching_FiberN	2421.71739	2361.18617	0.00032	*
All_MaxFracMatching_FiberNDivLength	147.57052	148.86977	0.67707	
All_MaxFracMatching_Unweighted	116.02174	116.22872	0.02504	
All_MaxMatching_FAMean	80.63155	82.61073	0.66396	
All_MaxMatching_FiberLengthMean	5167.33947	5083.62843	0.31059	
All_MaxMatching_FiberN	2416.37391	2357.39362	0.00037	*
All_MaxMatching_FiberNDivLength	147.14008	148.37032	0.69510	
All_MaxMatching_Unweighted	115.77391	115.96809	0.05570	
All_MinCutBalDivSum_FAMean	0.01998	0.02065	0.71427	
All_MinCutBalDivSum_FiberLengthMean	0.01107	0.01083	0.69973	
All_MinCutBalDivSum_FiberN	0.02507	0.02029	0.00001	*
All_MinCutBalDivSum_FiberNDivLength	0.03425	0.03078	0.12279	
All_MinCutBalDivSum_Unweighted	0.01315	0.01234	0.09039	
All_MinSpanningForest_FAMean	50.81293	47.94001	0.00001	*
All_MinSpanningForest_FiberLengthMean	2794.74279	2822.10418	0.00013	*
All_MinSpanningForest_FiberN	245.53913	245.08511	0.56512	
All_MinSpanningForest_FiberNDivLength	8.05322	8.04386	0.94140	
All_MinVertexCoverBinary_Unweighted	165.60870	166.48936	0.07032	
All_MinVertexCover_FAMean	51.22634	49.27939	0.00001	*

All_MinVertexCover_FiberLengthMean	5170.96696	5072.65831	0.23874	
All_MinVertexCover_FiberN	2424.14348	2360.04787	0.00010	*
All_MinVertexCover_FiberNDivLength	128.48641	128.07573	0.70058	
All_MinVertexCover_Unweighted	116.02609	116.21277	0.04726	
All_PGEigengap_FAMean	0.01891	0.01708	0.00376	*
All_PGEigengap_FiberLengthMean	0.01548	0.01480	0.37661	
All_PGEigengap_FiberN	0.02475	0.02033	0.00001	*
All_PGEigengap_FiberNDivLength	0.02404	0.01916	0.00001	*
All_PGEigengap_Unweighted	0.01757	0.01595	0.00442	*
All_Sum_FAMean	673.67412	617.76979	0.00001	*
All_Sum_FiberLengthMean	50198.24877	47824.57236	0.00472	*
All_Sum_FiberN	13167.07826	12564.14894	0.00001	*
All_Sum_FiberNDivLength	619.73687	597.48943	0.00001	*
All_Sum_Unweighted	1794.95652	1737.55319	0.00001	*
Left_AdjLMaxDivD_FAMean	1.58747	1.61084	0.01155	
Left_AdjLMaxDivD_FiberLengthMean	1.66269	1.66928	0.55075	
Left_AdjLMaxDivD_FiberN	2.55064	2.63591	0.01316	
Left_AdjLMaxDivD_FiberNDivLength	2.46922	2.49502	0.38718	
Left_AdjLMaxDivD_Unweighted	1.42162	1.41012	0.03060	
Left_HoffmanBound_FAMean	4.13962	4.16628	0.29466	
Left_HoffmanBound_FiberLengthMean	3.14236	3.16818	0.33950	
Left_HoffmanBound_FiberN	2.60284	2.59995	0.88033	
Left_HoffmanBound_FiberNDivLength	2.52273	2.50376	0.34043	
Left_HoffmanBound_Unweighted	4.31547	4.30642	0.70557	
Left_LogSpanningForestN_FAMean	161.03690	150.27264	0.00001	*
Left_LogSpanningForestN_FiberLengthMean	664.06115	660.91658	0.17431	
Left_LogSpanningForestN_FiberN	481.98760	479.06065	0.02835	
Left_LogSpanningForestN_FiberNDivLength	130.81017	128.39044	0.03790	
Left_LogSpanningForestN_Unweighted	287.57562	285.21020	0.04173	
Left_MaxFracMatching_FAMean	40.84437	41.80238	0.68549	
Left_MaxFracMatching_FiberLengthMean	2683.08115	2626.73527	0.22816	
Left_MaxFracMatching_FiberN	1199.56522	1194.31383	0.63858	
Left_MaxFracMatching_FiberNDivLength	75.23642	76.01364	0.63792	
Left_MaxFracMatching_Unweighted	59.02174	59.15426	0.02276	
Left_MaxMatching_FAMean	40.73101	41.60035	0.71104	
Left_MaxMatching_FiberLengthMean	2679.39613	2637.73582	0.36598	
Left_MaxMatching_FiberN	1197.97391	1193.95745	0.72028	
Left_MaxMatching_FiberNDivLength	75.00839	75.81179	0.62408	
Left_MaxMatching_Unweighted	58.79130	58.86170	0.20064	
Left_MinCutBalDivSum_FAMean	0.20453	0.21086	0.71731	
Left_MinCutBalDivSum_FiberLengthMean	0.14246	0.12181	0.00001	*
Left_MinCutBalDivSum_FiberN	0.09868	0.08273	0.00001	*
Left_MinCutBalDivSum_FiberNDivLength	0.18665	0.19754	0.62306	
Left_MinCutBalDivSum_Unweighted	0.13857	0.12295	0.00001	*
Left_MinSpanningForest_FAMean	25.19855	23.64144	0.00001	*
Left_MinSpanningForest_FiberLengthMean	1423.68813	1438.50545	0.00028	*
Left_MinSpanningForest_FiberN	127.06957	127.25532	0.86129	
Left_MinSpanningForest_FiberNDivLength	4.18575	4.20175	0.87349	
Left_MinVertexCoverBinary_Unweighted	83.51304	83.96809	0.11146	
Left_MinVertexCover_FAMean	25.57763	24.42983	0.00001	*

Left_MinVertexCover_FiberLengthMean	2683.63258	2628.92733	0.23810	
Left_MinVertexCover_FiberN	1201.44348	1193.03723	0.44260	
Left_MinVertexCover_FiberNDivLength	65.50938	65.48566	0.97350	
Left_MinVertexCover_Unweighted	59.02174	59.14362	0.03941	
Left_PGEigengap_FAMean	0.13999	0.12101	0.00001	*
Left_PGEigengap_FiberLengthMean	0.14328	0.12313	0.00001	*
Left_PGEigengap_FiberN	0.09276	0.07605	0.00001	*
Left_PGEigengap_FiberNDivLength	0.07073	0.05988	0.00001	*
Left_PGEigengap_Unweighted	0.12467	0.10642	0.00001	*
Left_Sum_FAMean	333.37366	304.91149	0.00001	*
Left_Sum_FiberLengthMean	25766.38164	24536.70024	0.00808	*
Left_Sum_FiberN	6538.80000	6291.28723	0.00001	*
Left_Sum_FiberNDivLength	308.66574	299.55206	0.00180	*
Left_Sum_Unweighted	907.06957	880.01064	0.00012	*
Right_AdjLMaxDivD_FAMean	1.54054	1.54133	0.93800	
Right_AdjLMaxDivD_FiberLengthMean	1.63079	1.65634	0.05224	
Right_AdjLMaxDivD_FiberN	2.66812	2.74516	0.06771	
Right_AdjLMaxDivD_FiberNDivLength	2.39111	2.38887	0.93985	
Right_AdjLMaxDivD_Unweighted	1.39936	1.38662	0.04221	
Right_HoffmanBound_FAMean	4.13168	4.12126	0.65194	
Right_HoffmanBound_FiberLengthMean	3.17576	3.22074	0.08033	
Right_HoffmanBound_FiberN	2.53296	2.48305	0.00274	*
Right_HoffmanBound_FiberNDivLength	2.54416	2.47185	0.00005	*
Right_HoffmanBound_Unweighted	4.30307	4.28104	0.31741	
Right_LogSpanningForestN_FAMean	160.17261	151.97412	0.00005	*
Right_LogSpanningForestN_FiberLengthMean	637.98932	634.33567	0.12353	
Right_LogSpanningForestN_FiberN	463.72405	459.96553	0.00579	*
Right_LogSpanningForestN_FiberNDivLength	125.57724	123.36773	0.03373	
Right_LogSpanningForestN_Unweighted	277.44846	274.92442	0.03312	
Right_MaxFracMatching_FAMean	39.83302	40.91191	0.62599	
Right_MaxFracMatching_FiberLengthMean	2475.52017	2429.06018	0.27746	
Right_MaxFracMatching_FiberN	1133.25652	1131.97340	0.90188	
Right_MaxFracMatching_FiberNDivLength	70.27104	72.36800	0.18407	
Right_MaxFracMatching_Unweighted	57.00435	57.07979	0.22364	
Right_MaxMatching_FAMean	39.69325	40.75250	0.63017	
Right_MaxMatching_FiberLengthMean	2472.27779	2429.38282	0.31079	
Right_MaxMatching_FiberN	1131.71304	1127.52128	0.68653	
Right_MaxMatching_FiberNDivLength	70.03999	71.94388	0.22804	
Right_MaxMatching_Unweighted	56.72174	56.82979	0.08738	
Right_MinCutBalDivSum_FAMean	0.19791	0.19350	0.77184	
Right_MinCutBalDivSum_FiberLengthMean	0.14165	0.11868	0.00001	*
Right_MinCutBalDivSum_FiberN	0.09754	0.08410	0.00001	*
Right_MinCutBalDivSum_FiberNDivLength	0.18125	0.18593	0.81709	
Right_MinCutBalDivSum_Unweighted	0.13393	0.11525	0.00001	*
Right_MinSpanningForest_FAMean	25.69248	24.41148	0.00008	*
Right_MinSpanningForest_FiberLengthMean	1365.19318	1377.32416	0.00707	*
Right_MinSpanningForest_FiberN	119.54783	119.06383	0.29718	
Right_MinSpanningForest_FiberNDivLength	3.99147	3.98268	0.89969	
Right_MinVertexCoverBinary_Unweighted	81.69565	82.21277	0.07856	
Right_MinVertexCover_FAMean	25.49721	24.70942	0.00019	*

Right_MinVertexCover_FiberLengthMean	2473.11664	2427.90233	0.29233	
Right_MinVertexCover_FiberN	1133.89565	1131.75532	0.83718	
Right_MinVertexCover_FiberNDivLength	60.96391	62.13464	0.09034	
Right_MinVertexCover_Unweighted	57.00870	57.07447	0.29182	
Right_PGEigengap_FAMean	0.13714	0.11390	0.00001	*
Right_PGEigengap_FiberLengthMean	0.14364	0.11894	0.00001	*
Right_PGEigengap_FiberN	0.09419	0.07578	0.00001	*
Right_PGEigengap_FiberNDivLength	0.07216	0.06037	0.00001	*
Right_PGEigengap_Unweighted	0.12001	0.09968	0.00001	*
Right_Sum_FAMean	330.57089	304.41951	0.00001	*
Right_Sum_FiberLengthMean	23845.34938	22753.02865	0.01223	
Right_Sum_FiberN	6284.90435	6008.05319	0.00001	*
Right_Sum_FiberNDivLength	294.60676	285.84877	0.00046	*
Right_Sum_Unweighted	864.30435	835.94681	0.00005	*

Table S5

In this table, we give the graph-theoretic parameters computed for the 463-vertex graphs. The table contains their arithmetic means in the male and female groups, and the corresponding p-values for group 0 (see the "Statistical analysis" subsection). The graph-parameters and the syntax of the data are defined in the main text. Significant differences ($p < 0.01$) are denoted with an asterisk in the last column.

Property	Female	Male	p-value	
All_AdjLMaxDivD_FAMean	2.15152	2.13711	0.32214	
All_AdjLMaxDivD_FiberLengthMean	2.34498	2.33964	0.79818	
All_AdjLMaxDivD_FiberN	5.17834	5.08521	0.21601	
All_AdjLMaxDivD_FiberNDivLength	5.09639	4.80319	0.00063	*
All_AdjLMaxDivD_Unweighted	1.88865	1.84785	0.00023	*
All_HoffmanBound_FAMean	3.61964	3.63864	0.29281	
All_HoffmanBound_FiberLengthMean	2.92690	2.95102	0.27306	
All_HoffmanBound_FiberN	2.25823	2.26733	0.51416	
All_HoffmanBound_FiberNDivLength	2.22839	2.24269	0.30350	
All_HoffmanBound_Unweighted	3.73096	3.72649	0.81057	
All_LeftRatio_FAMean	0.97770	0.97919	0.75271	
All_LeftRatio_FiberLengthMean	1.01124	1.01629	0.37793	
All_LeftRatio_FiberN	0.99320	1.00153	0.07359	
All_LeftRatio_FiberNDivLength	0.99524	1.00124	0.17321	
All_LeftRatio_Unweighted	1.00145	1.00583	0.24066	
All_LogSpanningForestN_FAMean	438.34886	412.50442	0.00033	*
All_LogSpanningForestN_FiberLengthMean	2305.78858	2315.37932	0.34655	
All_LogSpanningForestN_FiberN	1447.86881	1446.88135	0.85703	
All_LogSpanningForestN_FiberNDivLength	150.36225	143.99520	0.05704	
All_LogSpanningForestN_Unweighted	934.65818	939.07724	0.38738	
All_MaxFracMatching_FAMean	98.71274	96.32183	0.63384	
All_MaxFracMatching_FiberLengthMean	7956.88960	7886.29757	0.56900	
All_MaxFracMatching_FiberN	2429.66522	2363.79787	0.00004	*
All_MaxFracMatching_FiberNDivLength	140.61036	138.76925	0.69162	
All_MaxFracMatching_Unweighted	221.49565	223.32447	0.00011	*

All_MaxMatching_FAMean	98.57616	96.20753	0.63640	
All_MaxMatching_FiberLengthMean	7947.72364	7904.18602	0.72316	
All_MaxMatching_FiberN	2425.98261	2360.14894	0.00003	*
All_MaxMatching_FiberNDivLength	140.13846	138.40665	0.70987	
All_MaxMatching_Unweighted	221.25217	223.14894	0.00006	*
All_MinCutBalDivSum_FAMean	0.01041	0.00969	0.17424	
All_MinCutBalDivSum_FiberLengthMean	0.00811	0.00763	0.28949	
All_MinCutBalDivSum_FiberN	0.02422	0.01949	0.00001	*
All_MinCutBalDivSum_FiberNDivLength	0.02518	0.02013	0.00009	*
All_MinCutBalDivSum_Unweighted	0.00910	0.00828	0.01617	
All_MinSpanningForest_FAMean	97.40278	92.60970	0.00001	*
All_MinSpanningForest_FiberLengthMean	5338.18749	5382.67530	0.00627	*
All_MinSpanningForest_FiberN	479.43478	480.13830	0.65286	
All_MinSpanningForest_FiberNDivLength	18.73746	18.70539	0.88733	
All_MinVertexCoverBinary_Unweighted	274.94783	279.74468	0.00020	*
All_MinVertexCover_FAMean	88.27609	86.05404	0.00196	*
All_MinVertexCover_FiberLengthMean	7960.31876	7887.04893	0.55370	
All_MinVertexCover_FiberN	2430.06957	2363.65426	0.00003	*
All_MinVertexCover_FiberNDivLength	130.90128	129.49926	0.14031	
All_MinVertexCover_Unweighted	221.47826	223.32979	0.00010	*
All_PGEigengap_FAMean	0.01118	0.01018	0.20415	
All_PGEigengap_FiberLengthMean	0.00906	0.00891	0.84118	
All_PGEigengap_FiberN	0.01884	0.01559	0.01070	
All_PGEigengap_FiberNDivLength	0.01752	0.01408	0.00291	*
All_PGEigengap_Unweighted	0.01001	0.00924	0.26355	
All_Sum_FAMean	1011.72390	943.89996	0.00001	*
All_Sum_FiberLengthMean	72818.65816	70226.45899	0.03170	
All_Sum_FiberN	13522.65217	12957.94681	0.00001	*
All_Sum_FiberNDivLength	654.01169	634.54064	0.00004	*
All_Sum_Unweighted	2760.65217	2721.54255	0.06866	
Left_AdjLMaxDivD_FAMean	2.13581	2.13924	0.82156	
Left_AdjLMaxDivD_FiberLengthMean	2.27317	2.26801	0.78357	
Left_AdjLMaxDivD_FiberN	4.06052	4.22036	0.00752	*
Left_AdjLMaxDivD_FiberNDivLength	3.88928	3.92498	0.47520	
Left_AdjLMaxDivD_Unweighted	1.85707	1.81941	0.00037	*
Left_HoffmanBound_FAMean	3.72900	3.78125	0.02704	
Left_HoffmanBound_FiberLengthMean	2.96178	2.98489	0.35233	
Left_HoffmanBound_FiberN	2.50420	2.47733	0.11044	
Left_HoffmanBound_FiberNDivLength	2.46140	2.44273	0.25769	
Left_HoffmanBound_Unweighted	3.82171	3.83488	0.55227	
Left_LogSpanningForestN_FAMean	210.12642	196.40915	0.00019	*
Left_LogSpanningForestN_FiberLengthMean	1151.90236	1159.44304	0.14835	
Left_LogSpanningForestN_FiberN	721.86079	722.81452	0.75427	
Left_LogSpanningForestN_FiberNDivLength	72.52656	69.28217	0.13042	
Left_LogSpanningForestN_Unweighted	464.90819	468.28624	0.20386	
Left_MaxFracMatching_FAMean	48.53975	47.21580	0.60516	
Left_MaxFracMatching_FiberLengthMean	4032.23315	4001.12956	0.64087	
Left_MaxFracMatching_FiberN	1172.41304	1173.14894	0.94108	
Left_MaxFracMatching_FiberNDivLength	69.42651	69.81974	0.86649	
Left_MaxFracMatching_Unweighted	111.02174	112.06915	0.00022	*

Left_MaxMatching_FAMean	48.46705	47.17865	0.61382	
Left_MaxMatching_FiberLengthMean	4029.31001	4012.21426	0.79659	
Left_MaxMatching_FiberN	1168.78261	1172.34043	0.72673	
Left_MaxMatching_FiberNDivLength	69.12320	69.63778	0.82594	
Left_MaxMatching_Unweighted	110.73043	111.80851	0.00011	*
Left_MinCutBalDivSum_FAMean	0.09944	0.08854	0.05366	
Left_MinCutBalDivSum_FiberLengthMean	0.09034	0.07803	0.00002	*
Left_MinCutBalDivSum_FiberN	0.06837	0.05971	0.00001	*
Left_MinCutBalDivSum_FiberNDivLength	0.08119	0.07437	0.51027	
Left_MinCutBalDivSum_Unweighted	0.09033	0.07828	0.00001	*
Left_MinSpanningForest_FAMean	47.42016	44.98209	0.00001	*
Left_MinSpanningForest_FiberLengthMean	2684.63877	2718.61109	0.00029	*
Left_MinSpanningForest_FiberN	242.71304	244.21277	0.26029	
Left_MinSpanningForest_FiberNDivLength	9.52708	9.56275	0.79983	
Left_MinVertexCoverBinary_Unweighted	136.82609	139.48936	0.00028	*
Left_MinVertexCover_FAMean	43.23640	41.95796	0.00078	*
Left_MinVertexCover_FiberLengthMean	4034.19939	4001.84626	0.62665	
Left_MinVertexCover_FiberN	1172.68261	1172.98936	0.97547	
Left_MinVertexCover_FiberNDivLength	64.50611	65.12631	0.31298	
Left_MinVertexCover_Unweighted	111.02174	112.07447	0.00020	*
Left_PGEigengap_FAMean	0.08150	0.06923	0.00319	*
Left_PGEigengap_FiberLengthMean	0.08494	0.07246	0.00663	*
Left_PGEigengap_FiberN	0.06397	0.05295	0.00077	*
Left_PGEigengap_FiberNDivLength	0.04746	0.04080	0.00312	*
Left_PGEigengap_Unweighted	0.07018	0.05920	0.00189	*
Left_Sum_FAMean	494.72731	461.64225	0.00001	*
Left_Sum_FiberLengthMean	36823.20925	35652.14913	0.06912	
Left_Sum_FiberN	6711.51304	6483.65957	0.00001	*
Left_Sum_FiberNDivLength	325.29947	317.66272	0.01038	
Left_Sum_Unweighted	1381.98261	1368.69149	0.24506	
Right_AdjLMaxDivD_FAMean	2.04487	2.02697	0.29333	
Right_AdjLMaxDivD_FiberLengthMean	2.19527	2.21743	0.25616	
Right_AdjLMaxDivD_FiberN	4.29277	4.40447	0.13066	
Right_AdjLMaxDivD_FiberNDivLength	3.85170	3.79705	0.35469	
Right_AdjLMaxDivD_Unweighted	1.80761	1.77132	0.00152	*
Right_HoffmanBound_FAMean	3.62789	3.62380	0.83491	
Right_HoffmanBound_FiberLengthMean	2.96970	2.96886	0.96837	
Right_HoffmanBound_FiberN	2.39859	2.35006	0.00045	*
Right_HoffmanBound_FiberNDivLength	2.45102	2.39300	0.00005	*
Right_HoffmanBound_Unweighted	3.71467	3.70197	0.52197	
Right_LogSpanningForestN_FAMean	222.72004	210.78247	0.00395	*
Right_LogSpanningForestN_FiberLengthMean	1144.69241	1146.71072	0.72684	
Right_LogSpanningForestN_FiberN	717.84406	716.02759	0.58524	
Right_LogSpanningForestN_FiberNDivLength	72.40229	70.05104	0.26626	
Right_LogSpanningForestN_Unweighted	463.53301	464.48221	0.75133	
Right_MaxFracMatching_FAMean	50.04924	48.95794	0.65866	
Right_MaxFracMatching_FiberLengthMean	3910.77231	3870.86335	0.54062	
Right_MaxFracMatching_FiberN	1150.92174	1146.97872	0.70870	
Right_MaxFracMatching_FiberNDivLength	67.80675	68.08738	0.90588	
Right_MaxFracMatching_Unweighted	110.47826	111.23936	0.00309	*

Right_MaxMatching_FAMean	49.96835	48.87203	0.65625	
Right_MaxMatching_FiberLengthMean	3903.73000	3876.11184	0.66742	
Right_MaxMatching_FiberN	1150.87826	1144.00000	0.50989	
Right_MaxMatching_FiberNDivLength	67.68323	67.87734	0.93476	
Right_MaxMatching_Unweighted	110.25217	111.04255	0.00204	*
Right_MinCutBalDivSum_FAMean	0.10508	0.09217	0.01168	
Right_MinCutBalDivSum_FiberLengthMean	0.09559	0.08090	0.00001	*
Right_MinCutBalDivSum_FiberN	0.07388	0.06514	0.00001	*
Right_MinCutBalDivSum_FiberNDivLength	0.08568	0.07909	0.50488	
Right_MinCutBalDivSum_Unweighted	0.09328	0.07967	0.00001	*
Right_MinSpanningForest_FAMean	50.08262	47.75359	0.00005	*
Right_MinSpanningForest_FiberLengthMean	2647.49286	2658.31431	0.26500	
Right_MinSpanningForest_FiberN	238.13043	237.70213	0.67440	
Right_MinSpanningForest_FiberNDivLength	9.35118	9.32215	0.83349	
Right_MinVertexCoverBinary_Unweighted	137.79130	139.92553	0.00346	*
Right_MinVertexCover_FAMean	44.88732	43.91222	0.01455	
Right_MinVertexCover_FiberLengthMean	3912.35409	3870.75981	0.52310	
Right_MinVertexCover_FiberN	1151.06087	1147.00532	0.69964	
Right_MinVertexCover_FiberNDivLength	62.98171	63.52966	0.41129	
Right_MinVertexCover_Unweighted	110.46087	111.23936	0.00259	*
Right_PGEigengap_FAMean	0.08160	0.06725	0.00007	*
Right_PGEigengap_FiberLengthMean	0.08365	0.06956	0.00052	*
Right_PGEigengap_FiberN	0.06575	0.05389	0.00009	*
Right_PGEigengap_FiberNDivLength	0.05081	0.04228	0.00011	*
Right_PGEigengap_Unweighted	0.06961	0.05717	0.00008	*
Right_Sum_FAMean	506.02858	472.91635	0.00001	*
Right_Sum_FiberLengthMean	35355.01763	34004.03695	0.03615	
Right_Sum_FiberN	6466.53913	6209.79787	0.00001	*
Right_Sum_FiberNDivLength	312.21774	304.73958	0.00331	*
Right_Sum_Unweighted	1352.97391	1329.13830	0.05687	

Table S6

In this table, we give the graph-theoretic parameters computed for the 1015-vertex graphs. The table contains their arithmetic means in the male and female groups, and the corresponding p-values for group 0 (see the "Statistical analysis" subsection). The graph-parameters and the syntax of the data are defined in the main text. Significant differences ($p < 0.01$) are denoted with an asterisk in the last column.

Property	Female	Male	p-value	
All_AdjLMaxDivD_FAMean	3.26391	3.21057	0.03613	
All_AdjLMaxDivD_FiberLengthMean	3.60508	3.61334	0.81478	
All_AdjLMaxDivD_FiberN	10.24529	9.92755	0.07190	
All_AdjLMaxDivD_FiberNDivLength	10.08175	9.36632	0.00022	*
All_AdjLMaxDivD_Unweighted	2.81864	2.74618	0.00035	*
All_HoffmanBound_FAMean	3.12681	3.13308	0.67861	
All_HoffmanBound_FiberLengthMean	2.69885	2.72366	0.16335	
All_HoffmanBound_FiberN	2.18923	2.19336	0.71714	
All_HoffmanBound_FiberNDivLength	2.18250	2.19168	0.42770	

All_HoffmanBound_Unweighted	3.15010	3.16902	0.19317	
All_LeftRatio_FAMean	0.98504	0.98697	0.65952	
All_LeftRatio_FiberLengthMean	1.01722	1.02269	0.31705	
All_LeftRatio_FiberN	0.99283	1.00175	0.05057	
All_LeftRatio_FiberNDivLength	0.99580	1.00216	0.13961	
All_LeftRatio_Unweighted	1.00822	1.01172	0.34731	
All_LogSpanningForestN_FAMean	452.66618	401.59076	0.00002	*
All_LogSpanningForestN_FiberLengthMean	3998.62984	4045.89366	0.05608	
All_LogSpanningForestN_FiberN	2105.94128	2112.41461	0.55725	
All_LogSpanningForestN_FiberNDivLength	-350.40752	-376.52817	0.00003	*
All_LogSpanningForestN_Unweighted	1433.48889	1450.08490	0.11718	
All_MaxFracMatching_FAMean	375.55439	381.61057	0.65457	
All_MaxFracMatching_FiberLengthMean	12308.92247	12225.06692	0.65914	
All_MaxFracMatching_FiberN	2506.50877	2427.63298	0.00001	*
All_MaxFracMatching_FiberNDivLength	389.77109	392.09308	0.87595	
All_MaxFracMatching_Unweighted	415.21491	422.88298	0.00017	*
All_MaxMatching_FAMean	375.08500	373.49236	0.91172	
All_MaxMatching_FiberLengthMean	12306.61007	12267.82644	0.83636	
All_MaxMatching_FiberN	2505.11404	2426.30851	0.00001	*
All_MaxMatching_FiberNDivLength	382.10096	389.68108	0.63129	
All_MaxMatching_Unweighted	415.16964	413.96739	0.84640	
All_MinCutBalDivSum_FAMean	0.01274	0.01336	0.59086	
All_MinCutBalDivSum_FiberLengthMean	0.00602	0.00563	0.24543	
All_MinCutBalDivSum_FiberN	0.02374	0.01909	0.00001	*
All_MinCutBalDivSum_FiberNDivLength	0.03720	0.03516	0.51965	
All_MinCutBalDivSum_Unweighted	0.00668	0.00596	0.00518	*
All_MinSpanningForest_FAMean	201.14246	194.00690	0.00010	*
All_MinSpanningForest_FiberLengthMean	10810.54420	10938.08381	0.02244	
All_MinSpanningForest_FiberN	945.42105	957.31915	0.00215	*
All_MinSpanningForest_FiberNDivLength	42.55934	43.50234	0.00901	*
All_MinVertexCoverBinary_Unweighted	452.99123	464.13830	0.00021	*
All_MinVertexCover_FAMean	151.11374	148.75595	0.06900	
All_MinVertexCover_FiberLengthMean	12315.25393	12234.71947	0.67124	
All_MinVertexCover_FiberN	2508.62281	2429.99468	0.00001	*
All_MinVertexCover_FiberNDivLength	138.09749	136.42385	0.08085	
All_MinVertexCover_Unweighted	414.86161	422.88043	0.00009	*
All_PGEigengap_FAMean	0.00055	0.00060	0.84367	
All_PGEigengap_FiberLengthMean	0.00046	0.00056	0.68494	
All_PGEigengap_FiberN	0.00118	0.00120	0.97715	
All_PGEigengap_FiberNDivLength	0.00106	0.00105	0.98422	
All_PGEigengap_Unweighted	0.00049	0.00057	0.75560	
All_Sum_FAMean	1435.45723	1356.82420	0.00001	*
All_Sum_FiberLengthMean	100058.53295	97231.32300	0.07727	
All_Sum_FiberN	13702.51754	13165.47872	0.00001	*
All_Sum_FiberNDivLength	675.08211	658.32175	0.00051	*
All_Sum_Unweighted	3950.33333	3942.51064	0.81017	
Left_AdjLMaxDivD_FAMean	3.18888	3.15430	0.18683	
Left_AdjLMaxDivD_FiberLengthMean	3.45389	3.44831	0.86339	
Left_AdjLMaxDivD_FiberN	7.44018	7.75972	0.00670	*
Left_AdjLMaxDivD_FiberNDivLength	7.25256	7.28578	0.75531	

Left_AdjLMaxDivD_Unweighted	2.73200	2.66542	0.00095	*
Left_HoffmanBound_FAMean	3.18792	3.21815	0.10077	
Left_HoffmanBound_FiberLengthMean	2.72983	2.74135	0.56234	
Left_HoffmanBound_FiberN	2.39551	2.36640	0.05764	
Left_HoffmanBound_FiberNDivLength	2.37202	2.36850	0.80888	
Left_HoffmanBound_Unweighted	3.19577	3.21727	0.20726	
Left_LogSpanningForestN_FAMean	215.74379	190.37937	0.00005	*
Left_LogSpanningForestN_FiberLengthMean	2004.39019	2032.53459	0.03146	
Left_LogSpanningForestN_FiberN	1056.51036	1061.10443	0.46768	
Left_LogSpanningForestN_FiberNDivLength	-172.96633	-186.83057	0.00045	*
Left_LogSpanningForestN_Unweighted	718.69635	728.97009	0.07009	
Left_MaxFracMatching_FAMean	187.79143	190.67012	0.67347	
Left_MaxFracMatching_FiberLengthMean	6248.24855	6224.53571	0.81537	
Left_MaxFracMatching_FiberN	1211.57456	1215.34043	0.71012	
Left_MaxFracMatching_FiberNDivLength	193.58633	196.07530	0.73586	
Left_MaxFracMatching_Unweighted	207.90789	211.61170	0.00118	*
Left_MaxMatching_FAMean	187.56459	190.57398	0.65856	
Left_MaxMatching_FiberLengthMean	6246.36085	6246.80376	0.99648	
Left_MaxMatching_FiberN	1209.53509	1215.35106	0.56905	
Left_MaxMatching_FiberNDivLength	193.23385	195.74463	0.73407	
Left_MaxMatching_Unweighted	207.66667	211.58511	0.00055	*
Left_MinCutBalDivSum_FAMean	0.10408	0.10872	0.59997	
Left_MinCutBalDivSum_FiberLengthMean	0.05439	0.04836	0.00197	*
Left_MinCutBalDivSum_FiberN	0.04626	0.04202	0.00107	*
Left_MinCutBalDivSum_FiberNDivLength	0.21596	0.21436	0.94069	
Left_MinCutBalDivSum_Unweighted	0.05508	0.04783	0.00001	*
Left_MinSpanningForest_FAMean	97.84281	94.20558	0.00024	*
Left_MinSpanningForest_FiberLengthMean	5420.46856	5493.47367	0.01596	
Left_MinSpanningForest_FiberN	477.09649	483.20213	0.02006	
Left_MinSpanningForest_FiberNDivLength	21.48953	21.86841	0.08025	
Left_MinVertexCoverBinary_Unweighted	226.83333	232.63830	0.00034	*
Left_MinVertexCover_FAMean	74.33023	73.06766	0.06543	
Left_MinVertexCover_FiberLengthMean	6247.30024	6226.93453	0.84176	
Left_MinVertexCover_FiberN	1211.01316	1215.39894	0.65766	
Left_MinVertexCover_FiberNDivLength	68.29763	69.18052	0.16165	
Left_MinVertexCover_Unweighted	207.92982	211.65426	0.00104	*
Left_PGEigengap_FAMean	0.01291	0.01737	0.18451	
Left_PGEigengap_FiberLengthMean	0.01368	0.01822	0.20492	
Left_PGEigengap_FiberN	0.01193	0.01555	0.23713	
Left_PGEigengap_FiberNDivLength	0.00882	0.01175	0.19242	
Left_PGEigengap_Unweighted	0.01113	0.01460	0.22351	
Left_Sum_FAMean	706.86641	668.88465	0.00003	*
Left_Sum_FiberLengthMean	50905.16818	49686.48356	0.15828	
Left_Sum_FiberN	6797.47368	6589.18085	0.00006	*
Left_Sum_FiberNDivLength	335.85385	329.89072	0.04884	
Left_Sum_Unweighted	1989.76316	1994.22340	0.80307	
Right_AdjLMaxDivD_FAMean	3.13638	3.09093	0.11113	
Right_AdjLMaxDivD_FiberLengthMean	3.44271	3.49144	0.14483	
Right_AdjLMaxDivD_FiberN	7.87548	8.10827	0.10424	
Right_AdjLMaxDivD_FiberNDivLength	7.21299	7.11027	0.38977	

Right_AdjLMaxDivD_Unweighted	2.73516	2.66649	0.00147	*
Right_HoffmanBound_FAMean	3.12011	3.12021	0.99541	
Right_HoffmanBound_FiberLengthMean	2.72047	2.71627	0.80239	
Right_HoffmanBound_FiberN	2.32392	2.27174	0.00008	*
Right_HoffmanBound_FiberNDivLength	2.36632	2.30960	0.00001	*
Right_HoffmanBound_Unweighted	3.13240	3.14164	0.55805	
Right_LogSpanningForestN_FAMean	230.00215	205.04675	0.00017	*
Right_LogSpanningForestN_FiberLengthMean	1983.76491	2002.58051	0.15642	
Right_LogSpanningForestN_FiberN	1040.41771	1041.86173	0.81878	
Right_LogSpanningForestN_FiberNDivLength	-183.37842	-194.97616	0.00128	*
Right_LogSpanningForestN_Unweighted	707.75289	713.51596	0.32314	
Right_MaxFracMatching_FAMean	187.58059	190.72286	0.64031	
Right_MaxFracMatching_FiberLengthMean	6042.45994	5986.31966	0.56896	
Right_MaxFracMatching_FiberN	1183.68860	1167.45213	0.07792	
Right_MaxFracMatching_FiberNDivLength	192.17256	194.82239	0.72256	
Right_MaxFracMatching_Unweighted	207.22368	211.03191	0.00048	*
Right_MaxMatching_FAMean	187.38097	190.51859	0.63955	
Right_MaxMatching_FiberLengthMean	6043.98429	6006.54168	0.70074	
Right_MaxMatching_FiberN	1184.21930	1166.60638	0.05839	
Right_MaxMatching_FiberNDivLength	191.91345	194.56752	0.72161	
Right_MaxMatching_Unweighted	207.02632	211.02128	0.00023	*
Right_MinCutBalDivSum_FAMean	0.10814	0.11038	0.80060	
Right_MinCutBalDivSum_FiberLengthMean	0.05818	0.05098	0.00006	*
Right_MinCutBalDivSum_FiberN	0.04803	0.04336	0.00001	*
Right_MinCutBalDivSum_FiberNDivLength	0.23186	0.22678	0.82294	
Right_MinCutBalDivSum_Unweighted	0.05872	0.05119	0.00001	*
Right_MinSpanningForest_FAMean	103.42417	99.90568	0.00063	*
Right_MinSpanningForest_FiberLengthMean	5384.31437	5439.30295	0.07352	
Right_MinSpanningForest_FiberN	471.37719	478.13830	0.00337	*
Right_MinSpanningForest_FiberNDivLength	21.28508	21.93744	0.00392	*
Right_MinVertexCoverBinary_Unweighted	225.98246	231.21277	0.00179	*
Right_MinVertexCover_FAMean	76.57533	75.67481	0.20158	
Right_MinVertexCover_FiberLengthMean	6050.51258	5994.51322	0.56911	
Right_MinVertexCover_FiberN	1185.97807	1169.50000	0.07615	
Right_MinVertexCover_FiberNDivLength	66.21118	66.27928	0.90881	
Right_MinVertexCover_Unweighted	207.20614	211.31915	0.00018	*
Right_PGEigengap_FAMean	0.01491	0.00973	0.11019	
Right_PGEigengap_FiberLengthMean	0.01571	0.00988	0.08719	
Right_PGEigengap_FiberN	0.01257	0.00820	0.11014	
Right_PGEigengap_FiberNDivLength	0.00965	0.00642	0.12520	
Right_PGEigengap_Unweighted	0.01268	0.00812	0.09616	
Right_Sum_FAMean	716.58340	678.16757	0.00005	*
Right_Sum_FiberLengthMean	48496.80981	46948.77164	0.06732	
Right_Sum_FiberN	6560.14035	6312.01064	0.00001	*
Right_Sum_FiberNDivLength	322.67446	316.28095	0.01331	
Right_Sum_Unweighted	1933.52632	1922.39362	0.53559	
